# Supplementary material for: Association between pharmaceutical modulation of oestrogen in postmenopausal women in Sweden and death due to COVID-19: a cohort study
Source: BMJ Open. 2022 Jan 14;12(2):e053032. doi: 10.1136/bmjopen-2021-053032 (PMC8844968; doi:10.1136/bmjopen-2021-053032)
Supplement: Author's manuscript [file bmjopen-2021-053032.draft_revisions.pdf]

BMJ Open is committed to open peer review. As part of this commitment we make the peer review history of every article we publish publicly available.

When an article is published we post the peer reviewers' comments and the authors' responses online. We also post the versions of the paper that were used during peer review. These are the versions that the peer review comments apply to.

The versions of the paper that follow are the versions that were submitted during the peer review process. They are not the versions of record or the final published versions. They should not be cited or distributed as the published version of this manuscript.

BMJ Open is an open access journal and the full, final, typeset and author-corrected version of record of the manuscript is available on our site with no access controls, subscription charges or pay-per-view fees (http://bmjopen.bmj.com).

If you have any questions on BMJ Open's open peer review process please email info.bmjopen@bmj.com

BMJ Open

Pharmaceutical modulation of oestrogen during COVID-19 – a nationwide cohort study

Journal:	*BMJ Open*
Manuscript ID	bmjopen-2021-053032
Article Type:	Original research
Date Submitted by the Author:	03-May-2021
Complete List of Authors:	Sund, Malin; Umeå Universitet Medicinska fakulteten, Department of Surgical and Perioperative Sciences<br>Fonseca-Rodríguez, Osvaldo; Umeå Universitet Medicinska fakulteten, Department of Clinical Microbiology<br>Josefsson, Andreas; Umeå Universitet Medicinska fakulteten, Department of Surgical and Perioperative Sciences/ Urology; University of Gothenburg, Department of Urology<br>Welen, Karin; University of Gothenburg, Department of Urology<br>Fors Connolly, Anne-Marie; Umeå Universitet Medicinska fakulteten, Department of Clinical Microbiology
Keywords:	COVID-19, Sex steroids & HRT < DIABETES & ENDOCRINOLOGY, EPIDEMIOLOGY, Epidemiology < INFECTIOUS DISEASES

SCHOLARONE™
Manuscripts

I, the Submitting Author has the right to grant and does grant on behalf of all authors of the Work (as defined in the below author licence), an exclusive licence and/or a non-exclusive licence for contributions from authors who are: i) UK Crown employees; ii) where BMJ has agreed a CC-BY licence shall apply, and/or iii) in accordance with the terms applicable for US Federal Government officers or employees acting as part of their official duties; on a worldwide, perpetual, irrevocable, royalty-free basis to BMJ Publishing Group Ltd ("BMJ") its licensees and where the relevant Journal is co-owned by BMJ to the co-owners of the Journal, to publish the Work in this journal and any other BMJ products and to exploit all rights, as set out in our licence.

The Submitting Author accepts and understands that any supply made under these terms is made by BMJ to the Submitting Author unless you are acting as an employee on behalf of your employer or a postgraduate student of an affiliated institution which is paying any applicable article publishing charge ("APC") for Open Access articles. Where the Submitting Author wishes to make the Work available on an Open Access basis (and intends to pay the relevant APC), the terms of reuse of such Open Access shall be governed by a Creative Commons licence – details of these licences and which Creative Commons licence will apply to this Work are set out in our licence referred to above.

Other than as permitted in any relevant BMJ Author's Self Archiving Policies, I confirm this Work has not been accepted for publication elsewhere, is not being considered for publication elsewhere and does not duplicate material already published. I confirm all authors consent to publication of this Work and authorise the granting of this licence.

For peer review only

Pharmaceutical modulation of oestrogen during COVID-19 – a nationwide cohort study

Malin Sund MD PhD[1,2], Osvaldo Fonseca-Rodríguez DVM PhD[3], Andreas Josefsson MD PhD[4-6], Karin Welen PhD[6], Anne-Marie Fors Connolly MD PhD[3]*

1. Department of Surgical and Perioperative Sciences/ Surgery, Umeå University, Umeå, Sweden
2. Department of Surgery, University of Helsinki and Helsinki University Hospital, Helsinki, Finland
3. Department of Clinical Microbiology, Umeå University, Umeå, Sweden
4. Department of Surgical and Perioperative Sciences/ Urology, Umeå University, Umeå, Sweden
5. Wallenberg Center for Molecular Medicine, Umeå University, Umeå Sweden
6. Department of Urology, Sahlgrenska Center for Cancer Research, Institute of Clinical Sciences, Sahlgrenska Academy, University of Gothenburg, Gothenburg, Sweden

* Corresponding author:
Anne-Marie Fors Connolly
Department of Clinical Microbiology
Umeå University
901 85 Umeå
Sweden
E-mail: anne-marie.fors.connolly@umu.se

Number of words text: 1406

Number of words abstract: 298

For peer review only - http://bmjopen.bmj.com/site/about/guidelines.xhtml

Abstract

Objective: Determine if oestrogen augmentation decreases the risk of death following COVID-19.

Design: Nationwide registry-based study

Participants: Postmenopausal women between 50 and 80 years of age with verified COVID-19 were divided into three groups: 1) Women with previously diagnosed breast cancer and receiving endocrine therapy (decreased systemic oestrogen levels); 2) women receiving hormone replacement therapy (HRT; increased systemic oestrogen levels) and 3) control group not fulfilling requirements for group 1 or 2 (postmenopausal oestrogen levels).

Main outcome measures: The main outcome was death following COVID-19, and the exposure was pharmaceutical modulation of oestrogen levels. Adjustments were made for potential confounders such as age, annual disposable income (richest group as the reference category), highest level of education (primary, secondary and tertiary (reference)) and the weighted Charlson Comorbidity Index (wCCI).

Results: From a nationwide cohort consisting of 49,853 women diagnosed with COVID-19 between the 4th of February to 14th of September 2020 in Sweden, 16,693 were between 50 to 80 years of age. We included 14,685 women in the study with 11,923 (81%) in the control group, 227 (2%) women in group 1 and 2,535 (17%) women in group 2. The unadjusted odds ratio (OR) for death following COVID-19 was 2.35 (95% CI 1.51-3.65) for group 1 and 0.45 (0.34-0.6) for group 2. Only the adjusted OR for death remained significant for group 2 with OR 0.47 (0.34-0.63). The risk of death due to COVID-19 was significantly associated with: Age OR 1.15 (1.14-1.17); annual income (poorest 2.79 (1.96-3.97); poor 2.43 (1.71-3.46) and middle 1.64 (1.11-2.41)); education (primary 1.4 (1.07-1.81)) and wCCI 1.13 (1.1-1.16).

For peer review only - http://bmjopen.bmj.com/site/about/guidelines.xhtml

Conclusions: Oestrogen supplementation in post-menopausal women is associated with a decreased risk of dying from COVID-19 in this nationwide cohort study. These findings are limited by the retrospective and non-randomized design. Further randomized intervention trials are warranted.

For peer review only

Strengths and limitations of the study

- This study is based on all diagnosed COVID-19 patients in Sweden
- Swedish registry data is well-validated and due to historical registry data and cross-linkage with the registries of Statistics Sweden, the confounding and/or effect modifying effects of socioeconomic variables and comorbidities could be adjusted for
- It investigates the effect of pharmaceutical modulation of oestrogen in post-menopausal women on death due to COVID-19
- The findings are limited by the retrospective and non-randomized design.

For peer review only

For peer review only - http://bmjopen.bmj.com/site/about/guidelines.xhtml

Introduction

The coronavirus disease 2019 (COVID-19) pandemic has swept across the globe causing enormous strain on societies and health care systems. Although women are infected, they appear to be protected from poor outcome when compared to men even after adjustment for confounding risk factors[1 2]. Similar epidemiological findings have also been described for SARS-CoV and MERS-CoV infections [3 4 5]. This implies biological differences between the sexes in terms of sensitivity to severe COVID-19, and oestrogen has been identified as a potential therapeutic candidate.

The majority of breast cancer (BC) patients have oestrogen receptor (ER) positive cancer [6], and are usually given adjuvant endocrine therapy after surgery in order to reduce the risk of cancer recurrences, leading to *reduced* systemic oestrogen levels. On the other hand, systemic oestrogen levels are *augmented* in women taking hormone replacement therapy (HRT) for relieving menopausal symptoms [7]. We use the opposing effects of endocrine therapy in BC patients and HRT in modulating systemic oestrogen levels in postmenopausal women as a model to test the hypothesis whether increased oestrogen levels are protective towards COVID-19 death in a nationwide cohort.

For peer review only

For peer review only - http://bmjopen.bmj.com/site/about/guidelines.xhtml

Materials and methods

Patients and public involvement statement

All data from the Swedish registries were pseudonoymized and therefore patients were not involved in the study.

Participants and sources of data

The personal identification numbers (PINs) from all diagnosed COVID-19 individuals in Sweden (SmiNet) between the 1st of February to 14th of September 2020 were cross-linked with the LISA Register (Longitudinal integrated database for health insurance and labor marker studies) administered by Statistics Sweden; and the following healthcare registers administreed by the Swedish National Board of Health and Welfare: Patient; Cancer; Prescribed pharmaceutical and Causes of Death. Post-menopausal women between the ages of 50-80 years of age were stratified into three groups: *Oestrogen decrease (group 1):* BC as identified by international classification of diseases (ICD) version 10 code C50, and the following treatment: tamoxifen or fulvestrant (anatomical therapeutic chemical (ATC): L02BA01, L02BA03) or an aromatase inhibitor (AI; ATC L02BG03, L02BG04, and L02BG06). *Augmented oestrogen (group 2):* Drugs classified as HRT (ATC codes: G03CA03, G03CA04, G03CC07, G03CX01, G03FA, G03FB). All ATC codes for group 1 and 2 were identified from the Prescribed Pharmaceutical Register with at least two consecutive withdrawals and at least one time should be during the period 2019-07-01 - to the latest date. *Native oestrogen (control group):* No BC diagnosis, and no prescription of the above-mentioned pharmaceuticals at any time point during 2019 and 2020. Ethical permit was granted by the Swedish Ethical Review Authority.

Outcome, confounders and effect modifiers

The outcome was death due to COVID-19 as identified by the ICD-10 code U07 as the main or contributing cause of death from the Cause of Death Register. Potential confounders and effect modifiers were included in the model and consisted of the weighted Charlson comorbidity index (wCCI)[8], age at COVID-19 diagnosis, income (divided into quintiles with the richest group as the

For peer review only - http://bmjopen.bmj.com/site/about/guidelines.xhtml

reference) and education (primary, secondary and tertiary (=reference)). The wCCI was calculated using the Patient and Cancer Registers, and up to two months prior to the COVID-19 date in order not to include complications due to COVID-19 as a comorbidity. If there was no information regarding diagnosis codes required for wCCI scoring, the individual was assigned a wCCI of zero. Information regarding income and education was retrieved from the LISA-register.

Statistical methods

The distribution of continuous and categorical variables in the three groups was tested using ANOVA and the χ^2 test, respectively. Each variable was then analyzed with univariate logistic regression models, followed by a multivariable regression model to compare the control group with group 1 and 2, respectively, and adjusting for confounders. Descriptive analyses and logistic regression models were performed in R statistical software version 4.0.2 using *finalfit* package 1.0.2.

For peer review only

Results

Participants

During the study period a total of 49,853 women of all ages were diagnosed with COVID-19 in Sweden, and a total of 14,685 women between the ages of 50-80 years of age were included in our study (Figure 1). Characteristics of these groups are shown in Table 1. Individuals with decreased oestrogen due to adjuvant endocrine therapy for BC (group 1) were older with a higher comorbidity index. A larger proportion of women in group 2 (increased levels of systemic oestrogen due to HRT) had high income and tertiary level of education (Table 1).

Oestrogen augmentation protects against death due to COVID-19

Pharmaceutically decreasing systemic oestrogen levels increased the odds of dying due to COVID-19 (group 1; odds ratio (OR) 2.35 95% confidence intervals (CI) 1.51-3.65), but following adjustment for confounders this association was no longer significant (Figure 2). Interestingly, augmentation of systemic oestrogen levels decreased the odds of dying due to COVID-19 with OR 0.45 (95% CI: 0.34-0.6), which remained significant even after adjustment for confounders (0.47 (95% CI: 0.34-0.63)). As expected, higher age and wCCI increased the odds of dying due to COVID-19. For every year increase in age the odds of dying was 1.15 (95% CI: 1.14-1.17) and for every increase in wCCI the odds of dying was 1.13 (95% CI: 1.10-1.16) (Figure 2). Furthermore, low income and having only primary level education were also factors that increased the odds of dying due to COVID-19 (Figure 2).

For peer review only - http://bmjopen.bmj.com/site/about/guidelines.xhtml

Discussion

Principal findings

The major finding of this nationwide registry-based study is that pharmaceutically augmenting oestrogen levels is associated with decreased odds of death due to COVID-19.

Comparison with related studies

There are several possible biological explanations for the lower risk experienced by women. These include mechanisms directly involved in viral internalization and reproduction, where oestrogen has been shown to decrease expression of vital proteins such as ACE2 and TMPRSS2 [9 10], inherent sex-linked differences in the immune system and direct oestrogen effects[11]. Our findings are supported by *in vitro* studies where 17β-estradiol treatment reduced viral load of SARS-CoV-2 [9]. Previous experimental studies in mice on SARS-CoV have moreover shown that female mice were less susceptible to infection, and that this protection was lost upon oophorectomy thus indicating a direct protective role of oestrogen signalling [12]. Furthermore, Barh et al. showed using a multiomics approach on SARS-CoV-2 infected host interactome, proteome, transcriptome, and bibliome datasets that oestrogen modulation could be a potential therapeutic option in COVID-19 [13]. Our findings are further verified by a smaller study of women taking HRT (n = 439) that showed similar results with oestrogen augmentation being associated lower risk of COVID-19 death, although in that study the risk selection bias was more difficult to descern since the cohort was neither population-based nor adjusted for central confounders [14]. In our study the effect of increased systemic oestrogen levels on reducing the risk of COVID-19 death remained significant also after adjusting for education level and income, both factors known to influence COVID-19 outcome [15], which further supports the protective role of oestrogen in women.

The hypothetic inverse, worsening, effect of reduced systemic oestrogen levels in women with a previous BC receiving adjuvant endocrine therapy was initially signficant but not after adjusting for confounders. This population differs from the control group in that they all have been diagnosed with BC and it has been shown that patients, both men and women, with any cancer form are harder hit by COVID-19 [16]. However, BC patients were in a previous study shown to be healthier compared to the

For peer review only - http://bmjopen.bmj.com/site/about/guidelines.xhtml

background population in terms of ischemic cardiac disease and CCI [17], and the wCCI adjustments may therefore overcompensate for this cancer-related vulnerability. Although not significant, a trend towards worse outcome remained and thus a larger population of BC patients on endocrine therapy is likely needed to verify the finding. Thus, this study cannot exclude an increased risk for death from COVID-19 if systemic oestrogen levels are pharmaceutically decreased.

Strengths and limitations

Strengths of this study are that this is a nationwide cohort in a country with high COVID-19 incidence using well validated registry data. A weakness is that the level of oestrogen modulation cannot be exactly measured in each individual, and that the number of BC women on anti-oestrogen medication ended up being too small to show significance although there was a clear trend. The HRT group, however, proved large enough to show the clear protective effect.

Implications and conclusion

The present study shows an association between oestrogen levels and COVID-19 death. Consequently, drugs increasing oestrogen levels may have a role in therapeutic efforts to alleviate COVID-19 severity in post-menopausal women and could be studied in randomized control trials.

For peer review only

Acknowledgments:

We would like to thank Wolfgang Lohr for data management and Dr- Chloé Jacquet for helping us design Figure 2.

Funding:

This study was funded by:

AMFC: Central ALF-funding, Region Västerbotten (RV-836351), Base unit ALF-funding (RV-939769); Strategic Funding during 2020 from the Department of Clinical Microbiology, Umeå University; Stroke Research in Northern Sweden; and Molecular Infection Medicine Sweden (MIMS).

AJ: Knut and Alice Wallenberg Foundation

Contributors

MS and KW conceptualized the study. MS and AMFC designed the study. OFR and AMFC prepared the study data. OFR performed the statistical analysis. All authors contributed to interpretation of the results. MS wrote the first draft of the manuscript. All authors contributed to critical revision of the manuscript. All authors approved the final manuscript. AMFC is the guarantor of this study. The corresponding author attests that all authors meet the criteria for authorship and that all have been included.

Competing interests

All authors have completed the ICMJE uniform disclosure form at www.icmje.org/coi_disclosure.pdf and declare: no support from any organisation for the submitted work; no financial relationships with any organisations that might have an interest in the submitted work in the previous three years; no other relationships or activities that could appear to have influenced the submitted work.

Ethical approval

Ethical approval was obtained by the Swedish Ethical Review Authority (number 2020-02150)

For peer review only

Data sharing

The study protocol (R script) is available upon request. The study used secondary registry data which is regulated by the Public Access to Information and Secrecy Act (2009:400) and is protected by strict confidentiality. For the purpose of research though, after formal application to access personal data the responsible authority can grant access to data, though this is contingent on vetting by the Ethical Review Authority of Sweden, according to the Act (2003:460) concerning the Ethical Review of Research Involving Humans. This means that the aggregated registry data cannot be shared.

For peer review only

For peer review only - http://bmjopen.bmj.com/site/about/guidelines.xhtml

References

1. Williamson EJ, Walker AJ, Bhaskaran K, et al. Factors associated with COVID-19-related death using OpenSAFELY. *Nature* 2020;584(7821):430-36. doi: 10.1038/s41586-020-2521-4

2. Abate BB, Kassie AM, Kassaw MW, et al. Sex difference in coronavirus disease (COVID-19): a systematic review and meta-analysis. *BMJ Open* 2020;10(10):e040129. doi: 10.1136/bmjopen-2020-040129 [published Online First: 2020/10/09]

3. Alghamdi IG, Hussain, II, Almalki SS, et al. The pattern of Middle East respiratory syndrome coronavirus in Saudi Arabia: a descriptive epidemiological analysis of data from the Saudi Ministry of Health. *Int J Gen Med* 2014;7:417-23. doi: 10.2147/IJGM.S67061 [published Online First: 2014/09/05]

4. Leong HN, Earnest A, Lim HH, et al. SARS in Singapore--predictors of disease severity. *Ann Acad Med Singap* 2006;35(5):326-31. [published Online First: 2006/07/11]

5. Karlberg J, Chong DS, Lai WY. Do men have a higher case fatality rate of severe acute respiratory syndrome than women do? *Am J Epidemiol* 2004;159(3):229-31. doi: 10.1093/aje/kwh056 [published Online First: 2004/01/27]

6. Sung H, Ferlay J, Siegel RL, et al. Global cancer statistics 2020: GLOBOCAN estimates of incidence and mortality worldwide for 36 cancers in 185 countries. *CA Cancer J Clin* 2021 doi: 10.3322/caac.21660

7. Delgado BJ, Lopez-Ojeda W. Oestrogen. StatPearls. Treasure Island (FL)2021.

8. Ludvigsson JF, Appelros P, Askling J, et al. Adaptation of the Charlson Comorbidity Index for Register-Based Research in Sweden. *Clin Epidemiol* 2021;13:21-41. doi: 10.2147/CLEP.S282475

9. Lemes RMR, Costa AJ, Bartolomeo CS, et al. 17beta-estradiol reduces SARS-CoV-2 infection in vitro. *Physiol Rep* 2021;9(2):e14707. doi: 10.14814/phy2.14707

For peer review only - http://bmjopen.bmj.com/site/about/guidelines.xhtml

10. Stelzig KE, Canepa-Escaro F, Schiliro M, et al. Oestrogen regulates the expression of SARS-CoV-2 receptor ACE2 in differentiated airway epithelial cells. *Am J Physiol Lung Cell Mol Physiol* 2020;318(6):L1280-L81. doi: 10.1152/ajplung.00153.2020

11. Klein SL, Flanagan KL. Sex differences in immune responses. *Nat Rev Immunol* 2016;16(10):626-38. doi: 10.1038/nri.2016.90

12. Channappanavar R, Fett C, Mack M, et al. Sex-Based Differences in Susceptibility to Severe Acute Respiratory Syndrome Coronavirus Infection. *J Immunol* 2017;198(10):4046-53. doi: 10.4049/jimmunol.1601896 [published Online First: 2017/04/05]

13. Barh D, Tiwari S, Weener ME, et al. Multi-omics-based identification of SARS-CoV-2 infection biology and candidate drugs against COVID-19. *Comput Biol Med* 2020;126:104051. doi: 10.1016/j.compbiomed.2020.104051

14. Seeland U, Coluzzi F, Simmaco M, et al. Evidence for treatment with estradiol for women with SARS-CoV-2 infection. *BMC Med* 2020;18(1):369. doi: 10.1186/s12916-020-01851-z

15. Drefahl S, Wallace M, Mussino E, et al. A population-based cohort study of socio-demographic risk factors for COVID-19 deaths in Sweden. *Nat Commun* 2020;11(1):5097. doi: 10.1038/s41467-020-18926-3

16. Zhang H, Han H, He T, et al. Clinical Characteristics and Outcomes of COVID-19-Infected Cancer Patients: A Systematic Review and Meta-Analysis. *J Natl Cancer Inst* 2020 doi: 10.1093/jnci/djaa168

17. Wennstig AK, Wadsten C, Garmo H, et al. Long-term risk of ischemic heart disease after adjuvant radiotherapy in breast cancer: results from a large population-based cohort. *Breast Cancer Res* 2020;22(1):10. doi: 10.1186/s13058-020-1249-2

For peer review only

Figure legends

Figure 1: Flow chart of the study

Figure 2: Oestrogen augmentation is associated with decreased odds of dying due to COVID-19. Crude and adjusted logistic regression models. Statistical significance: ($p < 0.05$ *, $p < 0.01$ **, $p < 0.001$ ***). OR odds ratio; CI confidence intervals; wCCI weighted Charlson Comorbidity Index.

For peer review only

Table 1. Characteristics of the study population

**Variable**		**Native oestrogen (control group)**	**Oestrogen decrease (group 1)**	**Augmented oestrogen (group 2)**	**p-value**
**Total N (%)**		11,923 (81.2)	227 (1.5)	2,535 (17.3)	
**Age**	Mean (SD)	61.2 (8.3)	64.4 (8.9)	60.9 (7.7)	<0.001
**wCCI**	Mean (SD)	1.4 (2.4)	5.0 (3.3)	1.6 (2.5)	<0.001
**Income quintiles, n (%)**	Richest	3,422 (28.7)	64 (28.2)	937 (37.0)	<0.001
	Rich	2,743 (23.0)	42 (18.5)	605 (23.9)	
	Middle	2,120 (17.8)	35 (15.4)	404 (15.9)	
	Poor	1,703 (14.3)	47 (20.7)	334 (13.2)	
	Poorest	1,903 (16.0)	39 (17.2)	253 (10.0)	
	Missing	32 (0.3)	0 (0)	2 (0.1)	
**Education, n (%)**	Tertiary	4,186 (35.1)	82 (36.1)	1074 (42.4)	<0.001
	Secondary	5,609 (47.0)	97 (42.7)	1150 (45.4)	
	Primary	1,882 (15.8)	45 (19.8)	290 (11.4)	
	Missing	246 (2.1)	3 (1.3)	21 (0.8)	

wCCI: Weighted Charlson Comorbidity Index. SD standard deviation

For peer review only

For peer review only - http://bmjopen.bmj.com/site/about/guidelines.xhtml

Figure 1

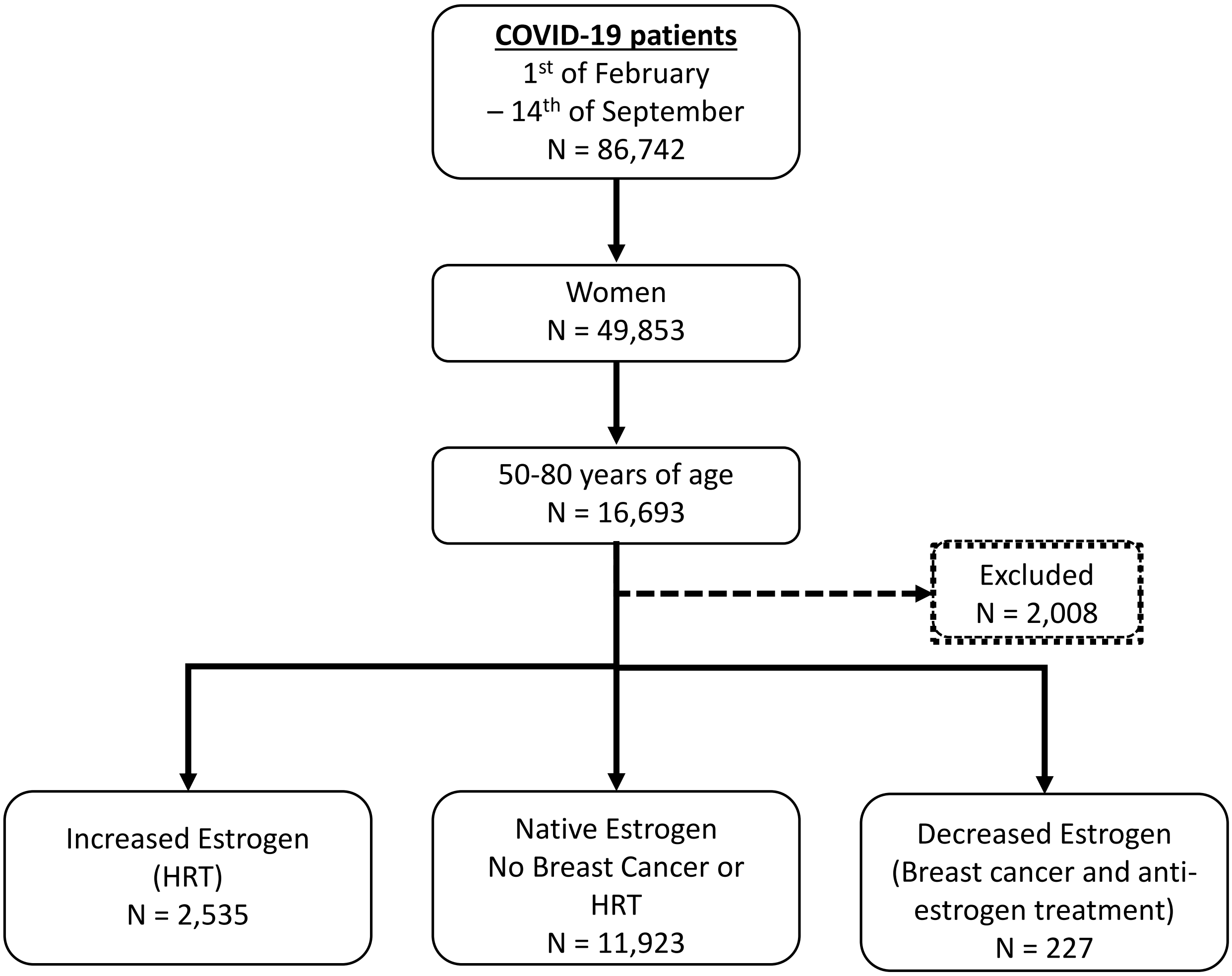
COVID-19 patients
1st of February
– 14th of September
N = 86,742
Women
N = 49,853
50-80 years of age
N = 16,693
Excluded
N = 2,008
Increased Estrogen
(HRT)
N = 2,535
Native Estrogen
No Breast Cancer or
HRT
N = 11,923
Decreased Estrogen
(Breast cancer and anti-
estrogen treatment)
N = 227

Figure 2

	Dead	Alive	Crude OR (95%CI)	Adjusted OR (95%CI)
**Estrogen Status, n (%)**				
Native estrogen	11377 (95.4)	546 (4.6)	-	-
Estrogen decrease	204 (89.9)	23 (10.1)	2.35 (1.51-3.65)***	1.21 (0.74-1.99)
Augmented estrogen	2 481 (97.9)	54 (2.1)	0.45 (0.34-0.60)***	0.47 (0.34-0.63)***
**Age in years**				
Mean (SD)	60.7 (7.9)	73.2 (6.4)	1.19 (1.18-1.21)***	1.15 (1.14-1.17)***
**wCCI**				
Mean (SD)	1.4 (2.4)	3.8 (3.1)	1.27 (1.25-1.30)***	1.13 (1.10-1.16)***
**Income, quintile, n (%)**				
Richest	4376 (98.9)	47 (1.1)	-	-
Rich	3346 (98.7)	44 (1.3)	1.22 (0.81-1.85)	1.14 (0.74-1.74)
Middle	2484 (97.1)	75 (2.9)	2.81 (1.95-4.06)***	1.64 (1.11-2.41)*
Poor	1886 (90.5)	198 (9.5)	9.77 (7.08-13.50)***	2.43 (1.71-3.46)***
Poorest	1937 (88.2)	258 (11.8)	12.40 (9.05-17.00)***	2.79 (1.96-3.97)***
**Education, n(%)**				
Tertiary	5228 (97.9)	114 (2.1)	-	-
Secondary	6615 (96.5)	241 (3.5)	1.67 (1.33-2.09)***	1.15 (0.90-1.47)
Primary	1988 (89.7)	229 (10.3)	5.28 (4.20-6.65)***	1.40 (1.07-1.81)**

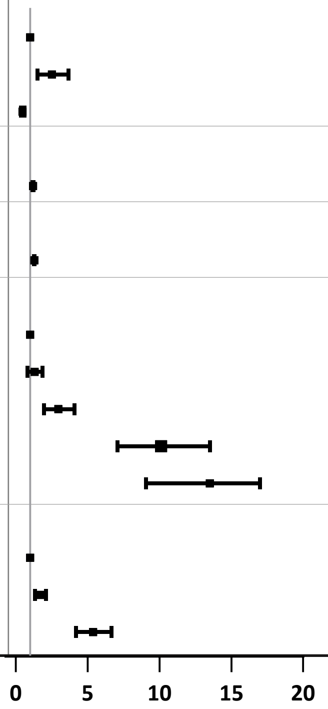

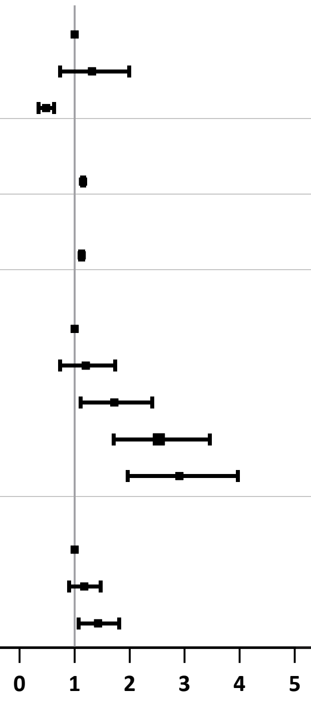

STROBE Statement—checklist of items that should be included in reports of observational studies

	Item No	Recommendation
**Title and abstract**	1	(*a*) Indicate the study's design with a commonly used term in the title or the abstract
		(*b*) Provide in the abstract an informative and balanced summary of what was done and what was found
**Introduction**		
Background/rationale	2	Explain the scientific background and rationale for the investigation being reported
Objectives	3	State specific objectives, including any prespecified hypotheses
**Methods**		
Study design	4	Present key elements of study design early in the paper
Setting	5	Describe the setting, locations, and relevant dates, including periods of recruitment, exposure, follow-up, and data collection
Participants	6	(*a*) *Cohort study*—Give the eligibility criteria, and the sources and methods of selection of participants. Describe methods of follow-up<br>*Case-control study*—Give the eligibility criteria, and the sources and methods of case ascertainment and control selection. Give the rationale for the choice of cases and controls<br>*Cross-sectional study*—Give the eligibility criteria, and the sources and methods of selection of participants
		(*b*) *Cohort study*—For matched studies, give matching criteria and number of exposed and unexposed<br>*Case-control study*—For matched studies, give matching criteria and the number of controls per case
Variables	7	Clearly define all outcomes, exposures, predictors, potential confounders, and effect modifiers. Give diagnostic criteria, if applicable
Data sources/ measurement	8*	For each variable of interest, give sources of data and details of methods of assessment (measurement). Describe comparability of assessment methods if there is more than one group
Bias	9	Describe any efforts to address potential sources of bias
Study size	10	Explain how the study size was arrived at
Quantitative variables	11	Explain how quantitative variables were handled in the analyses. If applicable, describe which groupings were chosen and why
Statistical methods	12	(*a*) Describe all statistical methods, including those used to control for confounding
		(*b*) Describe any methods used to examine subgroups and interactions
		(*c*) Explain how missing data were addressed
		(*d*) *Cohort study*—If applicable, explain how loss to follow-up was addressed<br>*Case-control study*—If applicable, explain how matching of cases and controls was addressed<br>*Cross-sectional study*—If applicable, describe analytical methods taking account of sampling strategy
		(*e*) Describe any sensitivity analyses

Continued on next page

For peer review only - http://bmjopen.bmj.com/site/about/guidelines.xhtml

**Results**		
Participants	13*	(a) Report numbers of individuals at each stage of study—eg numbers potentially eligible, examined for eligibility, confirmed eligible, included in the study, completing follow-up, and analysed
		(b) Give reasons for non-participation at each stage
		(c) Consider use of a flow diagram
Descriptive data	14*	(a) Give characteristics of study participants (eg demographic, clinical, social) and information on exposures and potential confounders
		(b) Indicate number of participants with missing data for each variable of interest
		(c) *Cohort study*—Summarise follow-up time (eg, average and total amount)
Outcome data	15*	*Cohort study*—Report numbers of outcome events or summary measures over time
		Case-control study—Report numbers in each exposure category, or summary measures of exposure
		Cross-sectional study—Report numbers of outcome events or summary measures
Main results	16	(*a*) Give unadjusted estimates and, if applicable, confounder-adjusted estimates and their precision (eg, 95% confidence interval). Make clear which confounders were adjusted for and why they were included
		(*b*) Report category boundaries when continuous variables were categorized
		(*c*) If relevant, consider translating estimates of relative risk into absolute risk for a meaningful time period
Other analyses	17	Report other analyses done—eg analyses of subgroups and interactions, and sensitivity analyses
**Discussion**		
Key results	18	Summarise key results with reference to study objectives
Limitations	19	Discuss limitations of the study, taking into account sources of potential bias or imprecision. Discuss both direction and magnitude of any potential bias
Interpretation	20	Give a cautious overall interpretation of results considering objectives, limitations, multiplicity of analyses, results from similar studies, and other relevant evidence
Generalisability	21	Discuss the generalisability (external validity) of the study results
**Other information**		
Funding	22	Give the source of funding and the role of the funders for the present study and, if applicable, for the original study on which the present article is based

*Give information separately for cases and controls in case-control studies and, if applicable, for exposed and unexposed groups in cohort and cross-sectional studies.

Note: An Explanation and Elaboration article discusses each checklist item and gives methodological background and published examples of transparent reporting. The STROBE checklist is best used in conjunction with this article (freely available on the Web sites of PLoS Medicine at http://www.plosmedicine.org/, Annals of Internal Medicine at http://www.annals.org/, and Epidemiology at http://www.epidem.com/). Information on the STROBE Initiative is available at www.strobe-statement.org.

For peer review only

Association between pharmaceutical modulation of oestrogen in postmenopausal women in Sweden with death due to COVID-19 – a cohort study

Journal:	*BMJ Open*
Manuscript ID	bmjopen-2021-053032.R1
Article Type:	Original research
Date Submitted by the Author:	27-Oct-2021
Complete List of Authors:	Sund, Malin; Umeå Universitet Medicinska fakulteten, Department of Surgical and Perioperative Sciences<br>Fonseca-Rodríguez, Osvaldo; Umeå Universitet Medicinska fakulteten, Department of Clinical Microbiology<br>Josefsson, Andreas; Umeå Universitet Medicinska fakulteten, Department of Surgical and Perioperative Sciences/ Urology; University of Gothenburg, Department of Urology<br>Welen, Karin; University of Gothenburg, Department of Urology<br>Fors Connolly, Anne-Marie; Umeå Universitet Medicinska fakulteten, Department of Clinical Microbiology
**Primary Subject Heading**:	Epidemiology
Secondary Subject Heading:	Infectious diseases, Oncology
Keywords:	COVID-19, Sex steroids & HRT < DIABETES & ENDOCRINOLOGY, EPIDEMIOLOGY, Epidemiology < INFECTIOUS DISEASES

I, the Submitting Author has the right to grant and does grant on behalf of all authors of the Work (as defined in the below author licence), an exclusive licence and/or a non-exclusive licence for contributions from authors who are: i) UK Crown employees; ii) where BMJ has agreed a CC-BY licence shall apply, and/or iii) in accordance with the terms applicable for US Federal Government officers or employees acting as part of their official duties; on a worldwide, perpetual, irrevocable, royalty-free basis to BMJ Publishing Group Ltd ("BMJ") its licensees and where the relevant Journal is co-owned by BMJ to the co-owners of the Journal, to publish the Work in this journal and any other BMJ products and to exploit all rights, as set out in our licence.

The Submitting Author accepts and understands that any supply made under these terms is made by BMJ to the Submitting Author unless you are acting as an employee on behalf of your employer or a postgraduate student of an affiliated institution which is paying any applicable article publishing charge ("APC") for Open Access articles. Where the Submitting Author wishes to make the Work available on an Open Access basis (and intends to pay the relevant APC), the terms of reuse of such Open Access shall be governed by a Creative Commons licence – details of these licences and which Creative Commons licence will apply to this Work are set out in our licence referred to above.

Other than as permitted in any relevant BMJ Author's Self Archiving Policies, I confirm this Work has not been accepted for publication elsewhere, is not being considered for publication elsewhere and does not duplicate material already published. I confirm all authors consent to publication of this Work and authorise the granting of this licence.

For peer review only

Association between pharmaceutical modulation of oestrogen in postmenopausal women in Sweden with death due to COVID-19 – a cohort study

Malin Sund MD PhD[1,2], Osvaldo Fonseca-Rodríguez DVM PhD[3], Andreas Josefsson MD PhD[4-6], Karin Welen PhD[6], Anne-Marie Fors Connolly MD PhD[3]*

1. Department of Surgical and Perioperative Sciences/ Surgery, Umeå University, Umeå, Sweden

2. Department of Surgery, University of Helsinki and Helsinki University Hospital, Helsinki, Finland

3. Department of Clinical Microbiology, Umeå University, Umeå, Sweden

4. Department of Surgical and Perioperative Sciences/ Urology, Umeå University, Umeå, Sweden

5. Wallenberg Center for Molecular Medicine, Umeå University, Umeå Sweden

6. Department of Urology, Sahlgrenska Center for Cancer Research, Institute of Clinical Sciences, Sahlgrenska Academy, University of Gothenburg, Gothenburg, Sweden

* Corresponding author:

Anne-Marie Fors Connolly

Department of Clinical Microbiology

Umeå University

901 85 Umeå

Sweden

E-mail: anne-marie.fors.connolly@umu.se

For peer review only - http://bmjopen.bmj.com/site/about/guidelines.xhtml

26 Number of words text: 1652

27 Number of words abstract: 297

For peer review only

For peer review only - http://bmjopen.bmj.com/site/about/guidelines.xhtml

Abstract

Objective: Determine whether augmentation of oestrogen in post-menopausal women decreases the risk of death following COVID-19.

Design: Nationwide study in Sweden based on registries from The Swedish Public Health Agency; Statistics Sweden (socioeconomical variables) and the National Board of Health and Welfare (Causes of death).

Participants: Postmenopausal women between 50 and 80 years of age with verified COVID-19.

Interventions: Pharmaceutical modulation of oestrogen as defined by (1) women with breast cancer receiving endocrine therapy (decreased systemic oestrogen levels); (2) postmenopausal hormone therapy (HT; increased systemic oestrogen levels) and (3) a control group (postmenopausal oestrogen levels). Adjustments were made for potential confounders such as age, annual disposable income (richest group as the reference category), highest level of education (primary, secondary and tertiary (reference)) and the weighted Charlson Comorbidity Index (wCCI).

Primary outcome measure: Death following COVID-19.

Results: From a nationwide cohort consisting of 49,853 women diagnosed with COVID-19 between February 4 and September 14, 2020 in Sweden, we included 14,685 women in the study with 11,923 (81%) in the control group, 227 (2%) women in group 1 and 2,535 (17%) women in group 2. The unadjusted odds ratio (OR) for death following COVID-19 was 2.35

For peer review only - http://bmjopen.bmj.com/site/about/guidelines.xhtml

For peer review only

(95% CI 1.51-3.65) for group 1 and 0.45 (0.34-0.6) for group 2. Only the adjusted OR for death remained significant for group 2 with OR 0.47 (0.34-0.63). Absolute risk (AR) of death was 4.6% for the control group vs 10.1% and 2.1%, for the decreased and increased oestrogen groups, respectively. The risk of death due to COVID-19 was significantly associated with: age, annual income, and education.

Conclusions: Oestrogen supplementation in post-menopausal women is associated with a decreased risk of dying from COVID-19 in this nationwide cohort study. These findings are limited by the retrospective and non-randomized design. Further randomized intervention trials are warranted.

Strengths and limitations of the study

- This study is based on all diagnosed COVID-19 patients in Sweden between February 1 and September 14, 2020
- Swedish registry data is well-validated and due to historical registry data and cross-linkage with the registries of Statistics Sweden, the confounding and/or effect-modifying effects of socioeconomic variables and comorbidities could be adjusted for
- The findings are limited by the retrospective and non-randomized design.
- Information regarding compliance to pharmaceutical modulation of oestrogen is missing
- Circulating oestrogen levels are not measured

For peer review only - http://bmjopen.bmj.com/site/about/guidelines.xhtml

Introduction

The coronavirus disease 2019 (COVID-19) pandemic has swept across the globe causing enormous strain on societies and health care systems. Although women are infected, they appear to be protected from poor outcomes when compared with men even after adjustment for confounding risk factors[1 2]. Similar epidemiological findings have also been described for SARS-CoV and MERS-CoV infections [3 4 5]. This implies biological differences between the sexes in terms of sensitivity to severe COVID-19, and oestrogen has been identified as a potential therapeutic candidate.

The majority of breast cancer (BC) patients have oestrogen receptor (ER)-positive cancer [6] and are usually given adjuvant endocrine therapy after surgery in order to reduce the risk of cancer recurrences, leading to *reduced* systemic oestrogen levels. On the other hand, systemic oestrogen levels are *augmented* in women taking postmenopausal hormone therapy (HT) to relieve menopausal symptoms [7]. In a nationwide cohort , we used the opposing effects of endocrine therapy in BC patients and women taking postmenopausal HT in modulating systemic oestrogen levels as a model to test the hypothesis that increased oestrogen levels are protective towards COVID-19 death.

For peer review only

Materials and methods

Patients and public involvement statement

All data from the Swedish registries were pseudonymised and therefore patients were not involved in the study.

Participants and sources of data

The personal identification numbers (PINs) from all diagnosed COVID-19 individuals in Sweden (SmiNet) between the February 1 and September 14, 2020 were cross-linked with the LISA Register (Longitudinal integrated database for health insurance and labour market studies) administered by Statistics Sweden; and the following healthcare registers administreed by the Swedish National Board of Health and Welfare: patient, cancer, prescribed pharmaceutical, and causes of death. Post-menopausal women 50-80 years of age were stratified into three groups as follows: Group 1, the *decreased oestrogen group,* included patients with BC as identified by the International Classification of Diseases (ICD) version 10 code C50, and the following treatments: tamoxifen or fulvestrant (anatomical therapeutic chemical (ATC): L02BA01, L02BA03) or an aromatase inhibitor (AI; ATC L02BG03, L02BG04, and L02BG06). Group 2, the *augmented oestrogen group,* included those patients treated with drugs classified as postmenopausal hormone therapy (ATC codes G03CA03, G03CA04, G03CC07, G03CX01, G03FA, and G03FB). All ATC codes for groups 1 and 2 were identified from the Prescribed Pharmaceutical Register with at least two consecutive withdrawals and at least one during the period extending from July 1, 2019 - to the latest date. Group 3, the *native oestrogen (control) group,* included patients with no BC diagnosis and no prescription of the above-mentioned pharmaceuticals at any time during 2019 and 2020. Ethical approval was granted by the Swedish Ethical Review Authority.

For peer review only - http://bmjopen.bmj.com/site/about/guidelines.xhtml

Outcome, confounders and effect modifiers

The outcome was death due to COVID-19, as identified by the ICD-10 code U07, as the main or contributing cause of death from the Cause of Death Register. Potential confounders and effect modifiers were included in the model and consisted of the weighted Charlson comorbidity index (wCCI)[8], age at COVID-19 diagnosis, income (divided into quintiles with the richest group as the reference) and education (primary, secondary and tertiary, which served as the reference). The wCCI was calculated using the Patient and Cancer Registers, up to 2 months prior to the COVID-19 date in order not to include complications due to COVID-19 as a comorbidity. If there was no information regarding diagnosis codes required for wCCI scoring, the individual was assigned a wCCI of zero. Information regarding income and education was retrieved from the LISA-register.

Statistical methods

The distributions of continuous and categorical variables in the three groups were tested using ANOVA and the χ^2 test, respectively. Each variable was then analysed with univariate logistic regression models, followed by a multivariable regression model to compare the control group with groups 1 and 2, respectively, and adjusting for confounders. Descriptive analyses and logistic regression models were performed using R statistical software version 4.0.2, using the *finalfit* package 1.0.2.

For peer review only

For peer review only - http://bmjopen.bmj.com/site/about/guidelines.xhtml

Results

Participants

During the study period a total of 49,853 women of all ages were diagnosed with COVID-19 in Sweden, and a total of 14,685 women aged 50-80 years were included in our study (figure 1). Characteristics of these groups are shown in table 1. Individuals with decreased oestrogen due to adjuvant endocrine therapy for BC (group 1) were older with a higher comorbidity index. A larger proportion of women in group 2 (increased levels of systemic oestrogen due to postmenopausal HT) had high income and a tertiary level of education (table 1).

Oestrogen augmentation protects against death due to COVID-19

Pharmaceutically decreasing systemic oestrogen levels increased the odds of dying due to COVID-19 (group 1: odds ratio [OR] 2.35, 95% confidence intervals [CI] 1.51 - 3.65), but following adjustment for confounders this association was no longer significant (figure 2). Interestingly, augmentation of systemic oestrogen levels decreased the odds of dying due to COVID-19, with OR 0.45 (95% CI 0.34 - 0.6), and this result remained significant even after adjustment for confounders (OR 0.47, 95% CI 0.34 - 0.63). The absolute risk (AR) of dying was 4.6% for the control group vs. 10.1% and 2.1% for the groups with decreased and increased oestrogen, respectively.

As expected, higher age and wCCI increased the odds of dying due to COVID-19. For every year increase in age the odds of dying were 1.15 (95% CI 1.14-1.17), and for every increase in wCCI the odds of dying were 1.13 (95% CI 1.10-1.16) (figure 2). Furthermore, low income and having only primary education were also factors that increased the odds of dying due to COVID-19 (figure 2).

For peer review only - http://bmjopen.bmj.com/site/about/guidelines.xhtml

Discussion

Principal findings

The major finding of this nationwide registry-based study is that pharmaceutical augmentation of oestrogen levels is associated with decreased odds of death due to COVID-19 in postmenopausal women.

Comparison with related studies

There are several possible biological explanations for the lower risk experienced by women. These include mechanisms directly involved in viral internalization and reproduction, where oestrogen has been shown to decrease expression of vital proteins such as ACE2 and TMPRSS2 [9-11], inherent sex-linked differences in the immune system, and direct oestrogen effects[12]. As an example, Kalidhindi et al have studied the effect of testosterone and oestrogen on ACE2 expression, a key cell entry for SARS-CoV-2 virus, using *in vitro* experiments on isolated human airway smooth muscle cells of male and female origin[13]. Most interestingly, they show that cells exposed to oestrogen and testosterone behave differently, as testosterone significantly upregulates ACE2 expression in cells from both sexes, whereas oestrogen downregulates ACE2[13]. ACE2 expression and differences in its expression in relation to sex could also be linked to the higher mortality in relation to hypertension, venous thromboembolism and SARS-CoV-2 infection between men and women [14]. The observed oestrogen induced reduction of ACE2 expression might however not necessarily translate into a reduction of ACE2 protein at the cell surface *in vivo* in all cell types. Our findings are also supported by *in vitro* studies where 17β-oestradiol treatment reduced SARS-CoV-2 viral load [9]. Previous experimental studies in mice on SARS-CoV have, moreover, shown that female mice are less susceptible to infection and that this protection was lost upon oophorectomy, thus indicating a direct protective role of oestrogen signalling [15]. Furthermore,

For peer review only - http://bmjopen.bmj.com/site/about/guidelines.xhtml

Barh et al., using a multiomics approach on SARS-CoV-2-infected host interactome, proteome, transcriptome, and bibliome datasets, demonstrated that oestrogen modulation could be a potential therapeutic option in COVID-19 [16]. Our results are in line with those by Seeland et al using real world evidence from multiple institutions and the TriNetX platform. They found by using propensity score matched analysis of data for women aged 50 and above with COVID-19 (n=439), that there was a survival benefit for oestradiol hormone-users versus non-users (OR 0.33 (95%CI 0.18-0.62)). Although based on a large real-world dataset the risk of selection bias was more difficult to discern since the cohort was neither population-based nor adjusted for central confounders although likely mitigated by the propensity score matched analysis [17]. In our study the effect of increased systemic oestrogen levels on reducing the risk of COVID-19 death remained significant after adjusting for education level and income, both factors known to influence COVID-19 outcome [18], further supporting the protective role of oestrogen in women.

The hypothetical inverse, a worsening effect of reduced systemic oestrogen levels in women with a previous BC receiving adjuvant endocrine therapy, was initially significant but not after adjusting for confounders. This population differs from the control group in that they all have been diagnosed with BC and it has been shown that patients, both men and women, with any cancer are harder hit by COVID-19 [19]. However, in a previous study BC patients were shown to be healthier compared with the background population in terms of ischemic cardiac disease and CCI [20], and the wCCI adjustments may therefore overcompensate for this cancer-related vulnerability. Although not significant, a trend towards worse outcome remained and thus a larger population of BC patients on endocrine therapy is likely needed to verify the finding. Thus, this study cannot exclude an increased risk of death from COVID-19 if systemic oestrogen levels are pharmaceutically decreased.

For peer review only - http://bmjopen.bmj.com/site/about/guidelines.xhtml

Strengths and limitations

The strengths of this study are that this is a nationwide cohort in a country with high COVID-19 incidence using well validated registry data. A weakness is that the level of oestrogen modulation cannot be exactly measured in each individual, and that the number of BC women on anti-oestrogen medication ended up being too small to show significance although there was a clear trend. The postmenopausal HT group, however, proved large enough to show the clear protective effect. A further limitation is that confounding factors such as body mass index (BMI), nutrition and smoking habits are not available in the nationwide registry data.

Implications and conclusion

This study shows an association between oestrogen levels and COVID-19 death. Consequently, drugs increasing oestrogen levels may have a role in therapeutic efforts to alleviate COVID-19 severity in post-menopausal women and could be studied in randomized control trials.

For peer review only

Acknowledgments:

We would like to thank Wolfgang Lohr for data management and Dr- Chloé Jacquet for helping us design figure 2.

Funding:

This study was funded by:

AMFC: Central ALF-funding, Region Västerbotten (RV-836351), Base unit ALF-funding (RV-939769); Strategic Funding during 2020 from the Department of Clinical Microbiology, Umeå University; Stroke Research in Northern Sweden; and Molecular Infection Medicine Sweden (MIMS).

AJ: the Knut and Alice Wallenberg Foundation

Contributors

MS and KW conceptualized the study. MS and AMFC designed the study, with input from KW, AJ and OFR. OFR and AMFC prepared the study data. OFR performed the statistical analysis. MS, KW, AJ, OFR and AMFC contributed to interpretation of the results. MS wrote the first draft of the manuscript. MS, KW, AJ, OFR and AMFC contributed to critical revision of the manuscript. MS, KW, AJ, OFR and AMFC approved the final manuscript. AMFC is the guarantor of this study. The corresponding author attests that all authors meet the criteria for authorship and that all have been included.

Competing interests

All authors have completed the ICMJE uniform disclosure form at www.icmje.org/coi_disclosure.pdf and declare: no support from any organisation for the submitted work, no financial relationships with any organisations that might have an interest

For peer review only

For peer review only - http://bmjopen.bmj.com/site/about/guidelines.xhtml

in the submitted work in the previous three years, and no other relationships or activities that could appear to have influenced the submitted work.

Ethical approval

Ethical approval was obtained by the Swedish Ethical Review Authority (number 2020-02150)

Data sharing

The study protocol (R script) is available upon request. The study used secondary registry data that are regulated by the Public Access to Information and Secrecy Act (2009:400) and are protected by strict confidentiality. For the purpose of research though, after formal application to access personal data the responsible authority can grant access to data, though this is contingent on vetting by the Ethical Review Authority of Sweden, according to the Act (2003:460) concerning the Ethical Review of Research Involving Humans. This means that the aggregated registry data cannot be shared.

For peer review only

References

1. Williamson EJ, Walker AJ, Bhaskaran K, et al. Factors associated with COVID-19-related death using OpenSAFELY. *Nature* 2020;584(7821):430-36. doi: 10.1038/s41586-020-2521-4

2. Abate BB, Kassie AM, Kassaw MW, et al. Sex difference in coronavirus disease (COVID-19): a systematic review and meta-analysis. *BMJ Open* 2020;10(10):e040129. doi: 10.1136/bmjopen-2020-040129 [published Online First: 2020/10/09]

3. Alghamdi IG, Hussain, II, Almalki SS, et al. The pattern of Middle East respiratory syndrome coronavirus in Saudi Arabia: a descriptive epidemiological analysis of data from the Saudi Ministry of Health. *Int J Gen Med* 2014;7:417-23. doi: 10.2147/IJGM.S67061 [published Online First: 2014/09/05]

4. Leong HN, Earnest A, Lim HH, et al. SARS in Singapore--predictors of disease severity. *Ann Acad Med Singap* 2006;35(5):326-31. [published Online First: 2006/07/11]

5. Karlberg J, Chong DS, Lai WY. Do men have a higher case fatality rate of severe acute respiratory syndrome than women do? *Am J Epidemiol* 2004;159(3):229-31. doi: 10.1093/aje/kwh056 [published Online First: 2004/01/27]

6. Sung H, Ferlay J, Siegel RL, et al. Global cancer statistics 2020: GLOBOCAN estimates of incidence and mortality worldwide for 36 cancers in 185 countries. *CA Cancer J Clin* 2021 doi: 10.3322/caac.21660

7. Delgado BJ, Lopez-Ojeda W. Estrogen. StatPearls. Treasure Island (FL)2021.

8. Ludvigsson JF, Appelros P, Askling J, et al. Adaptation of the Charlson Comorbidity Index for Register-Based Research in Sweden. *Clin Epidemiol* 2021;13:21-41. doi: 10.2147/CLEP.S282475

9. Lemes RMR, Costa AJ, Bartolomeo CS, et al. 17beta-estradiol reduces SARS-CoV-2 infection in vitro. *Physiol Rep* 2021;9(2):e14707. doi: 10.14814/phy2.14707

For peer review only - http://bmjopen.bmj.com/site/about/guidelines.xhtml

10. Stelzig KE, Canepa-Escaro F, Schiliro M, et al. Estrogen regulates the expression of SARS-CoV-2 receptor ACE2 in differentiated airway epithelial cells. *Am J Physiol Lung Cell Mol Physiol* 2020;318(6):L1280-L81. doi: 10.1152/ajplung.00153.2020

11. Young MJ, Clyne CD, Chapman KE. Endocrine aspects of ACE2 regulation: RAAS, steroid hormones and SARS-CoV-2. *J Endocrinol* 2020;247(2):R45-R62. doi: 10.1530/JOE-20-0260 [published Online First: 2020/09/24]

12. Klein SL, Flanagan KL. Sex differences in immune responses. *Nat Rev Immunol* 2016;16(10):626-38. doi: 10.1038/nri.2016.90

13. Kalidhindi RSR, Borkar NA, Ambhore NS, et al. Sex steroids skew ACE2 expression in human airway: a contributing factor to sex differences in COVID-19? *Am J Physiol Lung Cell Mol Physiol* 2020;319(5):L843-L47. doi: 10.1152/ajplung.00391.2020 [published Online First: 2020/10/01]

14. La Vignera S, Cannarella R, Condorelli RA, et al. Sex-Specific SARS-CoV-2 Mortality: Among Hormone-Modulated ACE2 Expression, Risk of Venous Thromboembolism and Hypovitaminosis D. *Int J Mol Sci* 2020;21(8) doi: 10.3390/ijms21082948 [published Online First: 2020/04/26]

15. Channappanavar R, Fett C, Mack M, et al. Sex-Based Differences in Susceptibility to Severe Acute Respiratory Syndrome Coronavirus Infection. *J Immunol* 2017;198(10):4046-53. doi: 10.4049/jimmunol.1601896 [published Online First: 2017/04/05]

16. Barh D, Tiwari S, Weener ME, et al. Multi-omics-based identification of SARS-CoV-2 infection biology and candidate drugs against COVID-19. *Comput Biol Med* 2020;126:104051. doi: 10.1016/j.compbiomed.2020.104051

17. Seeland U, Coluzzi F, Simmaco M, et al. Evidence for treatment with estradiol for women with SARS-CoV-2 infection. *BMC Med* 2020;18(1):369. doi: 10.1186/s12916-020-01851-z

For peer review only

18. Drefahl S, Wallace M, Mussino E, et al. A population-based cohort study of socio-demographic risk factors for COVID-19 deaths in Sweden. *Nat Commun* 2020;11(1):5097. doi: 10.1038/s41467-020-18926-3

19. Zhang H, Han H, He T, et al. Clinical Characteristics and Outcomes of COVID-19-Infected Cancer Patients: A Systematic Review and Meta-Analysis. *J Natl Cancer Inst* 2020 doi: 10.1093/jnci/djaa168

20. Wennstig AK, Wadsten C, Garmo H, et al. Long-term risk of ischemic heart disease after adjuvant radiotherapy in breast cancer: results from a large population-based cohort. *Breast Cancer Res* 2020;22(1):10. doi: 10.1186/s13058-020-1249-2

For peer review only

For peer review only - http://bmjopen.bmj.com/site/about/guidelines.xhtml

324 **Figure legends**

325 Figure 1: Flow chart of the study

326

327 Figure 2: Oestrogen augmentation is associated with decreased odds of dying due to COVID-
328 19. Crude and adjusted logistic regression models. Statistical significance: *p < 0.05, **p <
329 0.01, ***p < 0.001). OR, odds ratio; CI, confidence intervals; wCCI, weighted Charlson
330 Comorbidity Index.

For peer review only

For peer review only - http://bmjopen.bmj.com/site/about/guidelines.xhtml

Table 1. Characteristics of the study population

Variable		Native oestrogen (control group)	Decreased oestrogen (group 1)	Augmented oestrogen (group 2)	p-value
**Total N (%)**		11923 (81.2)	227 (1.5)	2535 (17.3)	
**Deaths**	No	11377 (95.4)	204 (89.9)	2481 (97.9)	<0.001
	Yes	546 (4.6)	23 (10.1)	54 (2.1)	
**Age**	Mean (SD)	61.2 (8.3)	64.4 (8.9)	60.9 (7.7)	<0.001
**wCCI**	Mean (SD)	1.4 (2.4)	5.0 (3.3)	1.6 (2.5)	<0.001
**Income quintiles, n (%)**	Richest	3422 (28.7)	64 (28.2)	937 (37.0)	<0.001
	Rich	2743 (23.0)	42 (18.5)	605 (23.9)	
	Middle	2120 (17.8)	35 (15.4)	404 (15.9)	
	Poor	1703 (14.3)	47 (20.7)	334 (13.2)	
	Poorest	1903 (16.0)	39 (17.2)	253 (10.0)	
	Missing	32 (0.3)	0 (0)	2 (0.1)	
**Education, n (%)**	Tertiary	4186 (35.1)	82 (36.1)	1074 (42.4)	<0.001
	Secondary	5609 (47.0)	97 (42.7)	1150 (45.4)	
	Primary	1882 (15.8)	45 (19.8)	290 (11.4)	
	Missing	246 (2.1)	3 (1.3)	21 (0.8)	

wCCI: Weighted Charlson Comorbidity Index. SD standard deviation

For peer review only

For peer review only - http://bmjopen.bmj.com/site/about/guidelines.xhtml

Figure 1

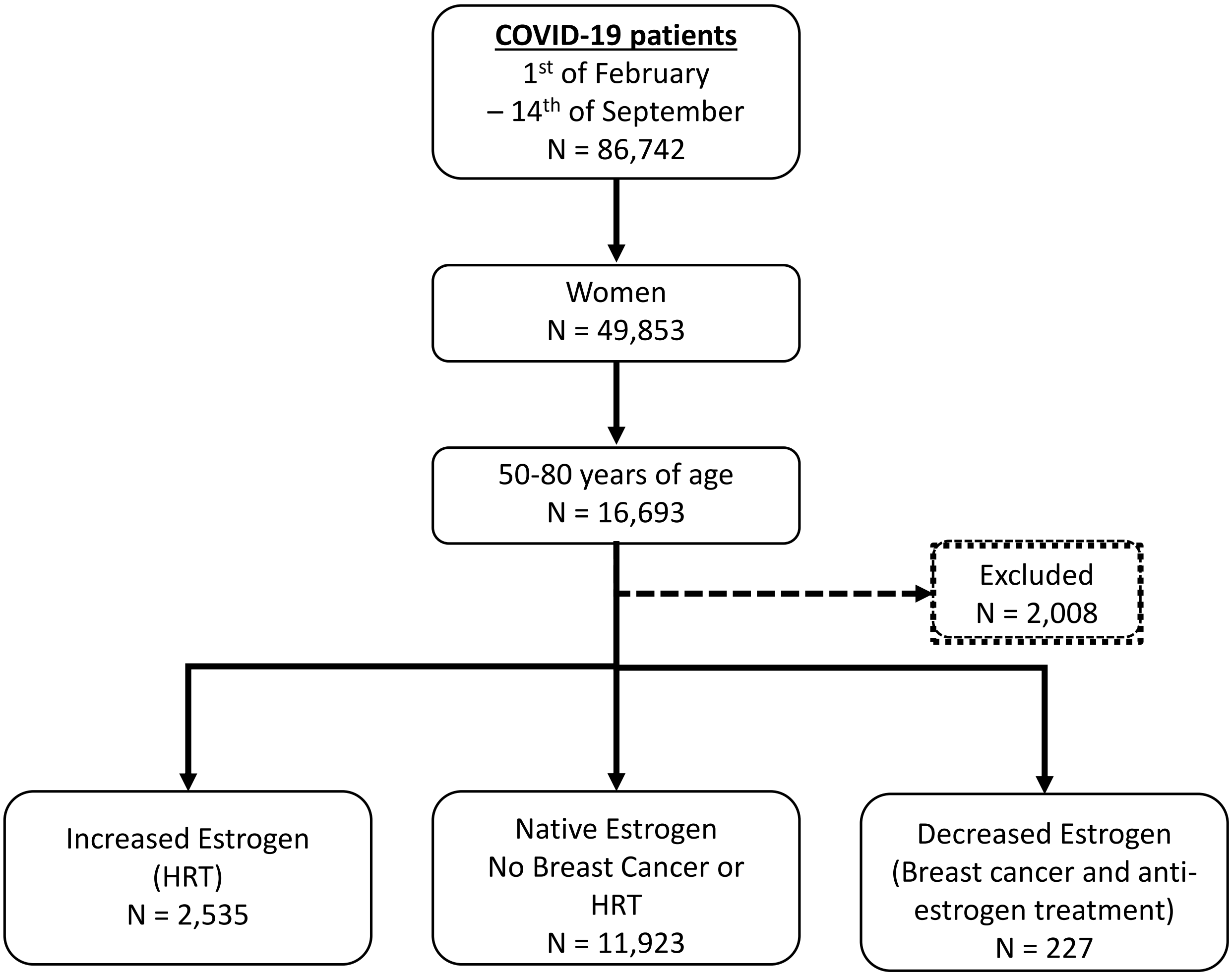
COVID-19 patients
1st of February
– 14th of September
N = 86,742
Women
N = 49,853
50-80 years of age
N = 16,693
Excluded
N = 2,008
Increased Estrogen
(HRT)
N = 2,535
Native Estrogen
No Breast Cancer or
HRT
N = 11,923
Decreased Estrogen
(Breast cancer and anti-
estrogen treatment)
N = 227

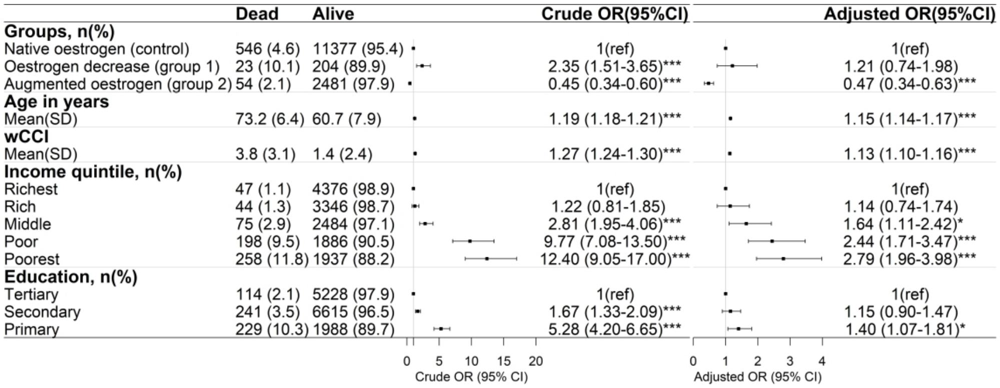

Figure 2

393x152mm (300 x 300 DPI)

STROBE Statement—Checklist of items that should be included in reports of ***cohort studies***

	Item No	Recommendation
**Title and abstract**	1	(*a*) Indicate the study's design with a commonly used term in the title or the abstract. *"Association between pharmaceutical modulation of oestrogen in postmenopausal women in Sweden with death due to COVID-19 – a cohort study". Page 1, line 1-2*
		(*b*) Provide in the abstract an informative and balanced summary of what was done and what was found. *Page 3-4, line 28-62*
**Introduction**		
Background/rationale	2	Explain the scientific background and rationale for the investigation being reported. *Page 5, lines 75-90.*
Objectives	3	State specific objectives, including any prespecified hypotheses. *Page 5, lines 87-90*
**Methods**		
Study design	4	Present key elements of study design early in the paper. *Page 6-7, lines 91-134*
Setting	5	Describe the setting, locations, and relevant dates, including periods of recruitment, exposure, follow-up, and data collection. *Page 6, lines 96-114*
Participants	6	(*a*) Give the eligibility criteria, and the sources and methods of selection of participants. Describe methods of follow-up. *Page 6, lines 96-114*
		(*b*) For matched studies, give matching criteria and number of exposed and unexposed. *Not applicable.*
Variables	7	Clearly define all outcomes, exposures, predictors, potential confounders, and effect modifiers. Give diagnostic criteria, if applicable. *Page 7, lines 116-126*
Data sources/ measurement	8*	For each variable of interest, give sources of data and details of methods of assessment (measurement). Describe comparability of assessment methods if there is more than one group. *Page 6-7, lines 91-134*
Bias	9	Describe any efforts to address potential sources of bias. *Not applicable.*
Study size	10	Explain how the study size was arrived at. *Page 6-7, lines 91-134*
Quantitative variables	11	Explain how quantitative variables were handled in the analyses. If applicable, describe which groupings were chosen and why. *Page 6-7, lines 91-134*
Statistical methods	12	(*a*) Describe all statistical methods, including those used to control for confounding. *Page 7, lines 128-134*
		(*b*) Describe any methods used to examine subgroups and interactions. *Page 6-7, lines 91-134*
		(*c*) Explain how missing data were addressed. *Case only method.*
		(*d*) If applicable, explain how loss to follow-up was addressed. *Not applicable.*
		(*e*) Describe any sensitivity analyses. *Not applicable.*
**Results**		
Participants	13*	(a) Report numbers of individuals at each stage of study—eg numbers potentially eligible, examined for eligibility, confirmed eligible, included in the study, completing follow-up, and analysed. *Page 8, lines 135-142*
		(b) Give reasons for non-participation at each stage. *Not applicable.*
		(c) Consider use of a flow diagram. *Figure 1.*
Descriptive data	14*	(a) Give characteristics of study participants (eg demographic, clinical, social) and information on exposures and potential confounders. *Page 8, lines 135-142, Table 1*

For peer review only - http://bmjopen.bmj.com/site/about/guidelines.xhtml

		(b) Indicate number of participants with missing data for each variable of interest. *Table 1.*
		(c) Summarise follow-up time (eg, average and total amount). *Not applicable.*
Outcome data	15*	Report numbers of outcome events or summary measures over time. *Page 8, lines 144-158.*
Main results	16	(*a*) Give unadjusted estimates and, if applicable, confounder-adjusted estimates and their precision (eg, 95% confidence interval). Make clear which confounders were adjusted for and why they were included. *Page 8, lines 144-158. Figure 2.*
		(*b*) Report category boundaries when continuous variables were categorized. *Not applicable.*
		(*c*) If relevant, consider translating estimates of relative risk into absolute risk for a meaningful time period. *Page 8, lines 150-152.*
Other analyses	17	Report other analyses done—eg analyses of subgroups and interactions, and sensitivity analyses. *Page 8, lines 154-158.*
**Discussion**		
Key results	18	Summarise key results with reference to study objectives. *Page 9, lines 160-163.*
Limitations	19	Discuss limitations of the study, taking into account sources of potential bias or imprecision. Discuss both direction and magnitude of any potential bias. *Page 11, lines 209-217.*
Interpretation	20	Give a cautious overall interpretation of results considering objectives, limitations, multiplicity of analyses, results from similar studies, and other relevant evidence. *Page 11, lines 219-223.*
Generalisability	21	Discuss the generalisability (external validity) of the study results. *Page 9-10, lines 165-207*
**Other information**		
Funding	22	Give the source of funding and the role of the funders for the present study and, if applicable, for the original study on which the present article is based. *Page 12, lines 228-234.*

*Give information separately for exposed and unexposed groups.

Note: An Explanation and Elaboration article discusses each checklist item and gives methodological background and published examples of transparent reporting. The STROBE checklist is best used in conjunction with this article (freely available on the Web sites of PLoS Medicine at http://www.plosmedicine.org/, Annals of Internal Medicine at http://www.annals.org/, and Epidemiology at http://www.epidem.com/). Information on the STROBE Initiative is available at http://www.strobe-statement.org.

For peer review only

For peer review only - http://bmjopen.bmj.com/site/about/guidelines.xhtml

BMJ Open

Association between pharmaceutical modulation of oestrogen in postmenopausal women in Sweden with death due to COVID-19 – a cohort study

Journal:	*BMJ Open*
Manuscript ID	bmjopen-2021-053032.R2
Article Type:	Original research
Date Submitted by the Author:	18-Nov-2021
Complete List of Authors:	Sund, Malin; Umeå Universitet Medicinska fakulteten, Department of Surgical and Perioperative Sciences<br>Fonseca-Rodríguez, Osvaldo; Umeå Universitet Medicinska fakulteten, Department of Clinical Microbiology<br>Josefsson, Andreas; Umeå Universitet Medicinska fakulteten, Department of Surgical and Perioperative Sciences/ Urology; University of Gothenburg, Department of Urology<br>Welen, Karin; University of Gothenburg, Department of Urology<br>Fors Connolly, Anne-Marie; Umeå Universitet Medicinska fakulteten, Department of Clinical Microbiology
**Primary Subject Heading**:	Epidemiology
Secondary Subject Heading:	Infectious diseases, Oncology
Keywords:	COVID-19, Sex steroids & HRT < DIABETES & ENDOCRINOLOGY, EPIDEMIOLOGY, Epidemiology < INFECTIOUS DISEASES

SCHOLARONE™
Manuscripts

I, the Submitting Author has the right to grant and does grant on behalf of all authors of the Work (as defined in the below author licence), an exclusive licence and/or a non-exclusive licence for contributions from authors who are: i) UK Crown employees; ii) where BMJ has agreed a CC-BY licence shall apply, and/or iii) in accordance with the terms applicable for US Federal Government officers or employees acting as part of their official duties; on a worldwide, perpetual, irrevocable, royalty-free basis to BMJ Publishing Group Ltd ("BMJ") its licensees and where the relevant Journal is co-owned by BMJ to the co-owners of the Journal, to publish the Work in this journal and any other BMJ products and to exploit all rights, as set out in our licence.

The Submitting Author accepts and understands that any supply made under these terms is made by BMJ to the Submitting Author unless you are acting as an employee on behalf of your employer or a postgraduate student of an affiliated institution which is paying any applicable article publishing charge ("APC") for Open Access articles. Where the Submitting Author wishes to make the Work available on an Open Access basis (and intends to pay the relevant APC), the terms of reuse of such Open Access shall be governed by a Creative Commons licence – details of these licences and which Creative Commons licence will apply to this Work are set out in our licence referred to above.

Other than as permitted in any relevant BMJ Author's Self Archiving Policies, I confirm this Work has not been accepted for publication elsewhere, is not being considered for publication elsewhere and does not duplicate material already published. I confirm all authors consent to publication of this Work and authorise the granting of this licence.

For peer review only

Association between pharmaceutical modulation of oestrogen in postmenopausal women in Sweden with death due to COVID-19 – a cohort study

Malin Sund MD PhD[1,2], Osvaldo Fonseca-Rodríguez DVM PhD[3], Andreas Josefsson MD PhD[4-6], Karin Welen PhD[6], Anne-Marie Fors Connolly MD PhD[3]*

1. Department of Surgical and Perioperative Sciences/ Surgery, Umeå University, Umeå, Sweden

2. Department of Surgery, University of Helsinki and Helsinki University Hospital, Helsinki, Finland

3. Department of Clinical Microbiology, Umeå University, Umeå, Sweden

4. Department of Surgical and Perioperative Sciences/ Urology, Umeå University, Umeå, Sweden

5. Wallenberg Center for Molecular Medicine, Umeå University, Umeå Sweden

6. Department of Urology, Sahlgrenska Center for Cancer Research, Institute of Clinical Sciences, Sahlgrenska Academy, University of Gothenburg, Gothenburg, Sweden

* Corresponding author:

Anne-Marie Fors Connolly

Department of Clinical Microbiology

Umeå University

901 85 Umeå

Sweden

E-mail: anne-marie.fors.connolly@umu.se

For peer review only

Number of words text: 1406

Number of words abstract: 298

For peer review only

For peer review only - http://bmjopen.bmj.com/site/about/guidelines.xhtml

Abstract

Objective: Determine whether augmentation of oestrogen in post-menopausal women decreases the risk of death following COVID-19.

Design: Nationwide registry-based study in Sweden based on registries from The Swedish Public Health Agency (all individuals that tested positive for SARS-CoV-2); Statistics Sweden (socioeconomical variables) and the National Board of Health and Welfare (Causes of death).

Participants: Postmenopausal women between 50 and 80 years of age with verified COVID-19.

Interventions: Pharmaceutical modulation of oestrogen as defined by (1) women with previously diagnosed breast cancer and receiving endocrine therapy (decreased systemic oestrogen levels); (2) women receiving hormone replacement therapy (HRT; increased systemic oestrogen levels) and (3) a control group not fulfilling requirements for group 1 or 2 (postmenopausal oestrogen levels). Adjustments were made for potential confounders such as age, annual disposable income (richest group as the reference category), highest level of education (primary, secondary and tertiary (reference)) and the weighted Charlson Comorbidity Index (wCCI).

Primary outcome measure: Death following COVID-19.

Results: From a nationwide cohort consisting of 49,853 women diagnosed with COVID-19 between February 4 and September 14, 2020 in Sweden, 16,693 were between 50 to 80 years

For peer review only - http://bmjopen.bmj.com/site/about/guidelines.xhtml

of age. We included 14,685 women in the study with 11,923 (81%) in the control group, 227 (2%) women in group 1 and 2,535 (17%) women in group 2. The unadjusted odds ratio (OR) for death following COVID-19 was 2.35 (95% CI 1.51-3.65) for group 1 and 0.45 (0.34-0.6) for group 2. Only the adjusted OR for death remained significant for group 2 with OR 0.47 (0.34-0.63). Absolute risk (AR) of death was 4.6% for the control group vs 10.1% and 2.1%, for the decreased and increased oestrogen groups, respectively. The risk of death due to COVID-19 was significantly associated with: age, OR 1.15 (1.14-1.17); annual income, poorest 2.79 [1.96-3.97], poor 2.43 [1.71-3.46] and middle 1.64 [1.11-2.41]; and education (primary 1.4 [1.07-1.81]) and wCCI 1.13 [1.1-1.16].

Conclusions: Oestrogen supplementation in post-menopausal women is associated with a decreased risk of dying from COVID-19 in this nationwide cohort study. These findings are limited by the retrospective and non-randomized design. Further randomized intervention trials are warranted.

For peer review only

Strengths and limitations of the study

- This study is based on all diagnosed COVID-19 patients in Sweden between February 1 and September 14, 2020
- Swedish registry data is well-validated and due to historical registry data and cross-linkage with the registries of Statistics Sweden, the confounding and/or effect-modifying effects of socioeconomic variables and comorbidities could be adjusted for
- The findings are limited by the retrospective and non-randomized design.
- Information regarding compliance to pharmaceutical modulation of oestrogen is missing

For peer review only - http://bmjopen.bmj.com/site/about/guidelines.xhtml

77 - Information about the exact duration of the postmenopausal hormone therapy (HT) was not available in the dataset
79 - Circulating oestrogen levels are not measured

For peer review only

For peer review only - http://bmjopen.bmj.com/site/about/guidelines.xhtml

Introduction

The coronavirus disease 2019 (COVID-19) pandemic has swept across the globe causing enormous strain on societies and health care systems. Although women are infected, they appear to be protected from poor outcomes when compared with men even after adjustment for confounding risk factors[1 2]. Similar epidemiological findings have also been described for SARS-CoV and MERS-CoV infections [3 4 5]. This implies biological differences between the sexes in terms of sensitivity to severe COVID-19, and oestrogen has been identified as a potential therapeutic candidate.

The majority of breast cancer (BC) patients have oestrogen receptor (ER)-positive cancer [6] and are usually given adjuvant endocrine therapy after surgery in order to reduce the risk of cancer recurrences, leading to *reduced* systemic oestrogen levels. On the other hand, systemic oestrogen levels are *augmented* in women taking postmenopausal hormone therapy (HT) to relieve menopausal symptoms [7]. In a nationwide cohort , we used the opposing effects of endocrine therapy in BC patients and women taking postmenopausal HT in modulating systemic oestrogen levels as a model to test the hypothesis that increased oestrogen levels are protective towards COVID-19 death.

For peer review only

For peer review only - http://bmjopen.bmj.com/site/about/guidelines.xhtml

Materials and methods

Patients and public involvement statement

All data from the Swedish registries were pseudonymised and therefore patients were not involved in the study.

Participants and sources of data

The personal identification numbers (PINs) from all diagnosed COVID-19 individuals in Sweden (SmiNet) between the February 1 and September 14, 2020 were cross-linked with the LISA Register (Longitudinal integrated database for health insurance and labour market studies) administered by Statistics Sweden; and the following healthcare registers administreed by the Swedish National Board of Health and Welfare: patient, cancer, prescribed pharmaceutical, and causes of death. Post-menopausal women 50-80 years of age were stratified into three groups as follows: Group 1, the *decreased oestrogen group,* included patients with BC as identified by the International Classification of Diseases (ICD) version 10 code C50, and the following treatments: tamoxifen or fulvestrant (anatomical therapeutic chemical (ATC): L02BA01, L02BA03) or an aromatase inhibitor (AI; ATC L02BG03, L02BG04, and L02BG06). Group 2, the *augmented oestrogen group,* included those patients treated with drugs classified as postmenopausal hormone therapy (ATC codes G03CA03, G03CA04, G03CC07, G03CX01, G03FA, and G03FB). All ATC codes for groups 1 and 2 were identified from the Prescribed Pharmaceutical Register with at least two consecutive withdrawals and at least one during the period extending from July 1, 2019 - to the latest date. Group 3, the *native oestrogen (control) group,* included patients with no BC diagnosis and no prescription of the above-mentioned pharmaceuticals at any time during 2019 and 2020. Ethical approval was granted by the Swedish Ethical Review Authority.

For peer review only - http://bmjopen.bmj.com/site/about/guidelines.xhtml

Outcome, confounders and effect modifiers

The outcome was death due to COVID-19, as identified by the ICD-10 code U07, as the main or contributing cause of death from the Cause of Death Register. Potential confounders and effect modifiers were included in the model and consisted of the weighted Charlson comorbidity index (wCCI)[8], age at COVID-19 diagnosis, income (divided into quintiles with the richest group as the reference) and education (primary, secondary and tertiary, which served as the reference). The wCCI was calculated using the Patient and Cancer Registers, up to 2 months prior to the COVID-19 date in order not to include complications due to COVID-19 as a comorbidity. If there was no information regarding diagnosis codes required for wCCI scoring, the individual was assigned a wCCI of zero. Information regarding income and education was retrieved from the LISA-register.

Statistical methods

The distributions of continuous and categorical variables in the three groups were tested using ANOVA and the χ^2 test, respectively. Each variable was then analysed with univariate logistic regression models, followed by a multivariable regression model to compare the control group with groups 1 and 2, respectively, and adjusting for confounders. The present study evaluates specific outcomes, and the odds ratio and p-values are adjusted for relevant confounders using the multivariate logistic regression model and there was no need to further adjust using the Bonferroni/Benjamini/FDR approach. Descriptive analyses and logistic regression models were performed using R statistical software version 4.0.2, using the *finalfit* package 1.0.2.

For peer review only - http://bmjopen.bmj.com/site/about/guidelines.xhtml

Results

Participants

During the study period a total of 49,853 women of all ages were diagnosed with COVID-19 in Sweden, and a total of 14,685 women aged 50-80 years were included in our study (figure 1). Characteristics of these groups are shown in table 1. Individuals with decreased oestrogen due to adjuvant endocrine therapy for BC (group 1) were older with a higher comorbidity index. A larger proportion of women in group 2 (increased levels of systemic oestrogen due to postmenopausal HT) had high income and a tertiary level of education (table 1).

Oestrogen augmentation protects against death due to COVID-19

Pharmaceutically decreasing systemic oestrogen levels increased the odds of dying due to COVID-19 (group 1: odds ratio [OR] 2.35, 95% confidence intervals [CI] 1.51 - 3.65), but following adjustment for confounders this association was no longer significant (figure 2). Interestingly, augmentation of systemic oestrogen levels decreased the odds of dying due to COVID-19, with OR 0.45 (95% CI 0.34 - 0.6), and this result remained significant even after adjustment for confounders (OR 0.47, 95% CI 0.34 - 0.63). The absolute risk (AR) of dying was 4.6% for the control group vs. 10.1% and 2.1% for the groups with decreased and increased oestrogen, respectively.

As expected, higher age and wCCI increased the odds of dying due to COVID-19. For every year increase in age the odds of dying were 1.15 (95% CI 1.14-1.17), and for every increase in wCCI the odds of dying were 1.13 (95% CI 1.10-1.16) (figure 2). Furthermore, low income and having only primary education were also factors that increased the odds of dying due to COVID-19 (figure 2).

For peer review only - http://bmjopen.bmj.com/site/about/guidelines.xhtml

Discussion

Principal findings

The major finding of this nationwide registry-based study is that pharmaceutical augmentation of oestrogen levels is associated with decreased odds of death due to COVID-19 in postmenopausal women.

Comparison with related studies

There are several possible biological explanations for the lower risk experienced by women. These include mechanisms directly involved in viral internalization and reproduction, where oestrogen has been shown to decrease expression of vital proteins such as ACE2 and TMPRSS2 [9-11], inherent sex-linked differences in the immune system, and direct oestrogen effects[12]. As an example, Kalidhindi et al have studied the effect of testosterone and oestrogen on ACE2, a key cell entry for SARS-CoV-2 virus, using *in vitro* experiments on isolated human airway smooth muscle cells of male and female origin[13]. Most interestingly, they show that cells exposed to oestrogen and testosterone behave differently, as testosterone significantly upregulates ACE2 expression in cells from both sexes, whereas oestrogen downregulates ACE2[13]. ACE2 expression and differences in its expression in relation to sex could also be linked to the higher mortality in relation to hypertension, venous thromboembolism and SARS-CoV-2 infection between men and women [14]. Our findings are also supported by *in vitro* studies where 17β-oestradiol treatment reduced SARS-CoV-2 viral load [9]. Previous experimental studies in mice on SARS-CoV have, moreover, shown that female mice are less susceptible to infection and that this protection was lost upon oophorectomy, thus indicating a direct protective role of oestrogen signalling [15]. Furthermore, Barh et al., using a multiomics approach on SARS-CoV-2-infected host interactome, proteome, transcriptome, and bibliome datasets, demonstrated that oestrogen modulation

For peer review only - http://bmjopen.bmj.com/site/about/guidelines.xhtml

could be a potential therapeutic option in COVID-19 [16]. Our results are in line with those by Seeland et al using real world evidence from multiple institutions and the TriNetX platform. They found by using propensity score matched analysis of data for women aged 50 and above with COVID-19 (n=439), that there was a survival benefit for oestradiol hormone-users versus non-users (OR 0.33 (95%CI 0.18-0.62)). Although based on a large real-world dataset the risk of selection bias was more difficult to discern since the cohort was neither population-based nor adjusted for central confounders although likely mitigated by the propensity score matched analysis [17]. In our study the effect of increased systemic oestrogen levels on reducing the risk of COVID-19 death remained significant after adjusting for education level and income, both factors known to influence COVID-19 outcome [18], further supporting the protective role of oestrogen in women. Adjusting for income and education is important as we have previously shown the how these affect the risk of dying due to COVID-19 in Sweden [19].

The hypothetical inverse, a worsening effect of reduced systemic oestrogen levels in women with a previous BC receiving adjuvant endocrine therapy, was initially significant but not after adjusting for confounders. This population differs from the control group in that they all have been diagnosed with BC and it has been shown that patients, both men and women, with any cancer are harder hit by COVID-19 [20]. However, in a previous study BC patients were shown to be healthier compared with the background population in terms of ischemic cardiac disease and CCI [21], and the wCCI adjustments may therefore overcompensate for this cancer-related vulnerability. Although not significant, a trend towards worse outcome remained and thus a larger population of BC patients on endocrine therapy is likely needed to verify the finding. Thus, this study cannot exclude an increased risk of death from COVID-19 if systemic oestrogen levels are pharmaceutically decreased.

For peer review only - http://bmjopen.bmj.com/site/about/guidelines.xhtml

Strengths and limitations

The strengths of this study are that this is a nationwide cohort in a country with high COVID-19 incidence using well validated registry data. A weakness is that the level of oestrogen modulation cannot be exactly measured in each individual, and that the number of BC women on anti-oestrogen medication ended up being too small to show significance although there was a clear trend. Furthermore, we do not have data on the exact duration of postmenopausal HT for the individuals. The postmenopausal HT group, however, proved large enough to show the clear protective effect. A further limitation is that confounding factors such as body mass index (BMI), nutrition and smoking habits are not available in the nationwide registry data.

Implications and conclusion

This study shows an association between oestrogen levels and COVID-19 death. Consequently, drugs increasing oestrogen levels may have a role in therapeutic efforts to alleviate COVID-19 severity in post-menopausal women and could be studied in randomized control trials.

For peer review only

Acknowledgments:

We would like to thank Wolfgang Lohr for data management and Dr- Chloé Jacquet for helping us design figure 2.

Funding:

This study was funded by:

AMFC: Central ALF-funding, Region Västerbotten (RV-836351), Base unit ALF-funding (RV-939769); Strategic Funding during 2020 from the Department of Clinical Microbiology, Umeå University; Stroke Research in Northern Sweden; and Molecular Infection Medicine Sweden (MIMS).

AJ: the Knut and Alice Wallenberg Foundation

Contributors

MS and KW conceptualized the study. MS and AMFC designed the study. OFR and AMFC prepared the study data. OFR performed the statistical analysis. MS, OFR, KW, AJ and AMFC all contributed to interpretation of the results. MS wrote the first draft of the manuscript. MS, OFR, KW, AJ and AMFC contributed to critical revision of the manuscript. MS, OFR, KW, AJ and AMFC approved the final manuscript. AMFC is the guarantor of this study. The corresponding author attests that all authors meet the criteria for authorship and that all have been included.

Competing interests

All authors have completed the ICMJE uniform disclosure form at www.icmje.org/coi_disclosure.pdf and declare: no support from any organisation for the submitted work, no financial relationships with any organisations that might have an interest

For peer review only - http://bmjopen.bmj.com/site/about/guidelines.xhtml

in the submitted work in the previous three years, and no other relationships or activities that could appear to have influenced the submitted work.

Ethical approval

Ethical approval was obtained by the Swedish Ethical Review Authority (number 2020-02150)

Data sharing

The study protocol (R script) is available upon request. The study used secondary registry data that are regulated by the Public Access to Information and Secrecy Act (2009:400) and are protected by strict confidentiality. For the purpose of research though, after formal application to access personal data the responsible authority can grant access to data, though this is contingent on vetting by the Ethical Review Authority of Sweden, according to the Act (2003:460) concerning the Ethical Review of Research Involving Humans. This means that the aggregated registry data cannot be shared.

For peer review only

References

1. Williamson EJ, Walker AJ, Bhaskaran K, et al. Factors associated with COVID-19-related death using OpenSAFELY. *Nature* 2020;584(7821):430-36. doi: 10.1038/s41586-020-2521-4
2. Abate BB, Kassie AM, Kassaw MW, et al. Sex difference in coronavirus disease (COVID-19): a systematic review and meta-analysis. *BMJ Open* 2020;10(10):e040129. doi: 10.1136/bmjopen-2020-040129 [published Online First: 2020/10/09]
3. Alghamdi IG, Hussain, II, Almalki SS, et al. The pattern of Middle East respiratory syndrome coronavirus in Saudi Arabia: a descriptive epidemiological analysis of data from the Saudi Ministry of Health. *Int J Gen Med* 2014;7:417-23. doi: 10.2147/IJGM.S67061 [published Online First: 2014/09/05]
4. Leong HN, Earnest A, Lim HH, et al. SARS in Singapore--predictors of disease severity. *Ann Acad Med Singap* 2006;35(5):326-31. [published Online First: 2006/07/11]
5. Karlberg J, Chong DS, Lai WY. Do men have a higher case fatality rate of severe acute respiratory syndrome than women do? *Am J Epidemiol* 2004;159(3):229-31. doi: 10.1093/aje/kwh056 [published Online First: 2004/01/27]
6. Sung H, Ferlay J, Siegel RL, et al. Global cancer statistics 2020: GLOBOCAN estimates of incidence and mortality worldwide for 36 cancers in 185 countries. *CA Cancer J Clin* 2021 doi: 10.3322/caac.21660
7. Delgado BJ, Lopez-Ojeda W. Estrogen. StatPearls. Treasure Island (FL)2021.
8. Ludvigsson JF, Appelros P, Askling J, et al. Adaptation of the Charlson Comorbidity Index for Register-Based Research in Sweden. *Clin Epidemiol* 2021;13:21-41. doi: 10.2147/CLEP.S282475
9. Lemes RMR, Costa AJ, Bartolomeo CS, et al. 17beta-estradiol reduces SARS-CoV-2 infection in vitro. *Physiol Rep* 2021;9(2):e14707. doi: 10.14814/phy2.14707
10. Stelzig KE, Canepa-Escaro F, Schiliro M, et al. Estrogen regulates the expression of SARS-CoV-2 receptor ACE2 in differentiated airway epithelial cells. *Am J Physiol Lung Cell Mol Physiol* 2020;318(6):L1280-L81. doi: 10.1152/ajplung.00153.2020
11. Young MJ, Clyne CD, Chapman KE. Endocrine aspects of ACE2 regulation: RAAS, steroid hormones and SARS-CoV-2. *J Endocrinol* 2020;247(2):R45-R62. doi: 10.1530/JOE-20-0260 [published Online First: 2020/09/24]
12. Klein SL, Flanagan KL. Sex differences in immune responses. *Nat Rev Immunol* 2016;16(10):626-38. doi: 10.1038/nri.2016.90
13. Kalidhindi RSR, Borkar NA, Ambhore NS, et al. Sex steroids skew ACE2 expression in human airway: a contributing factor to sex differences in COVID-19? *Am J Physiol Lung Cell Mol Physiol* 2020;319(5):L843-L47. doi: 10.1152/ajplung.00391.2020 [published Online First: 2020/10/01]
14. La Vignera S, Cannarella R, Condorelli RA, et al. Sex-Specific SARS-CoV-2 Mortality: Among Hormone-Modulated ACE2 Expression, Risk of Venous Thromboembolism and Hypovitaminosis D. *Int J Mol Sci* 2020;21(8) doi: 10.3390/ijms21082948 [published Online First: 2020/04/26]
15. Channappanavar R, Fett C, Mack M, et al. Sex-Based Differences in Susceptibility to Severe Acute Respiratory Syndrome Coronavirus Infection. *J Immunol* 2017;198(10):4046-53. doi: 10.4049/jimmunol.1601896 [published Online First: 2017/04/05]
16. Barh D, Tiwari S, Weener ME, et al. Multi-omics-based identification of SARS-CoV-2 infection biology and candidate drugs against COVID-19. *Comput Biol Med* 2020;126:104051. doi: 10.1016/j.compbiomed.2020.104051
17. Seeland U, Coluzzi F, Simmaco M, et al. Evidence for treatment with estradiol for women with SARS-CoV-2 infection. *BMC Med* 2020;18(1):369. doi: 10.1186/s12916-020-01851-z

For peer review only - http://bmjopen.bmj.com/site/about/guidelines.xhtml

18. Drefahl S, Wallace M, Mussino E, et al. A population-based cohort study of socio-demographic risk factors for COVID-19 deaths in Sweden. *Nat Commun* 2020;11(1):5097. doi: 10.1038/s41467-020-18926-3
19. Gustafsson PE, San Sebastian M, Fonseca-Rodriguez O, et al. Inequitable impact of infection: social gradients in severe COVID-19 outcomes among all confirmed SARS-CoV-2 cases during the first pandemic wave in Sweden. *J Epidemiol Community Health* 2021 doi: 10.1136/jech-2021-216778 [published Online First: 2021/09/17]
20. Zhang H, Han H, He T, et al. Clinical Characteristics and Outcomes of COVID-19-Infected Cancer Patients: A Systematic Review and Meta-Analysis. *J Natl Cancer Inst* 2020 doi: 10.1093/jnci/djaa168
21. Wennstig AK, Wadsten C, Garmo H, et al. Long-term risk of ischemic heart disease after adjuvant radiotherapy in breast cancer: results from a large population-based cohort. *Breast Cancer Res* 2020;22(1):10. doi: 10.1186/s13058-020-1249-2

For peer review only

For peer review only - http://bmjopen.bmj.com/site/about/guidelines.xhtml

Figure legends

Figure 1: Flow chart of the study

Figure 2: Oestrogen augmentation is associated with decreased odds of dying due to COVID-19. Crude and adjusted logistic regression models. Statistical significance: *$p < 0.05$, **$p < 0.01$, ***$p < 0.001$). OR, odds ratio; CI, confidence intervals; wCCI, weighted Charlson Comorbidity Index.

For peer review only

For peer review only - http://bmjopen.bmj.com/site/about/guidelines.xhtml

Table 1. Characteristics of the study population

Variable		Native oestrogen (control group)	Decreased oestrogen (group 1)	Augmented oestrogen (group 2)	p-value
**Total N (%)**		11923 (81.2)	227 (1.5)	2535 (17.3)	
**Deaths**	No	11377 (95.4)	204 (89.9)	2481 (97.9)	<0.001
	Yes	546 (4.6)	23 (10.1)	54 (2.1)	
**Age**	Mean (SD)	61.2 (8.3)	64.4 (8.9)	60.9 (7.7)	<0.001
**wCCI**	Mean (SD)	1.4 (2.4)	5.0 (3.3)	1.6 (2.5)	<0.001
**Income quintiles, n (%)**	Richest	3422 (28.7)	64 (28.2)	937 (37.0)	<0.001
	Rich	2743 (23.0)	42 (18.5)	605 (23.9)	
	Middle	2120 (17.8)	35 (15.4)	404 (15.9)	
	Poor	1703 (14.3)	47 (20.7)	334 (13.2)	
	Poorest	1903 (16.0)	39 (17.2)	253 (10.0)	
	Missing	32 (0.3)	0 (0)	2 (0.1)	
**Education, n (%)**	Tertiary	4186 (35.1)	82 (36.1)	1074 (42.4)	<0.001
	Secondary	5609 (47.0)	97 (42.7)	1150 (45.4)	
	Primary	1882 (15.8)	45 (19.8)	290 (11.4)	
	Missing	246 (2.1)	3 (1.3)	21 (0.8)	

wCCI: Weighted Charlson Comorbidity Index. SD standard deviation

For peer review only

For peer review only - http://bmjopen.bmj.com/site/about/guidelines.xhtml

Figure 1

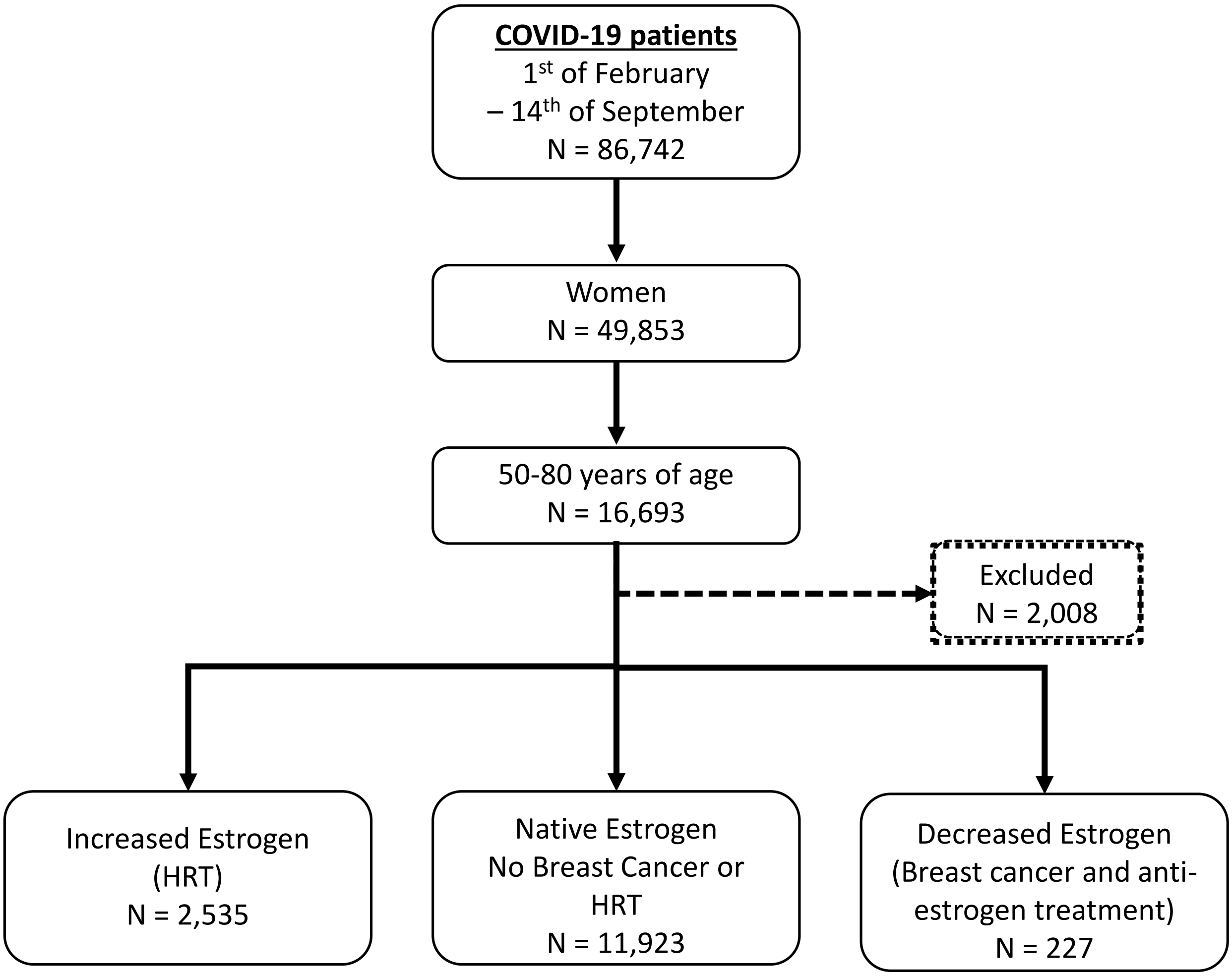
COVID-19 patients
1st of February
– 14th of September
N = 86,742
Women
N = 49,853
50-80 years of age
N = 16,693
Excluded
N = 2,008
Increased Estrogen
(HRT)
N = 2,535
Native Estrogen
No Breast Cancer or
HRT
N = 11,923
Decreased Estrogen
(Breast cancer and anti-
estrogen treatment)
N = 227

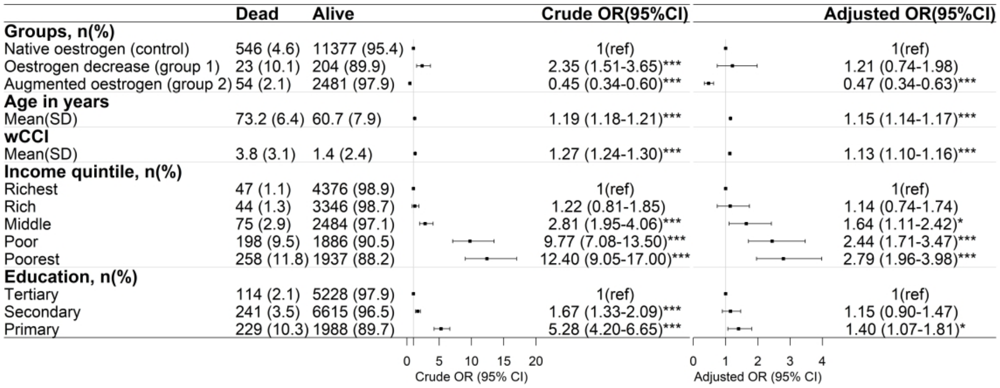

	Dead	Alive	Crude OR(95%CI)	Adjusted OR(95%CI)
**Groups, n(%)**				
Native oestrogen (control)	546 (4.6)	11377 (95.4)	1(ref)	1(ref)
Oestrogen decrease (group 1)	23 (10.1)	204 (89.9)	2.35 (1.51-3.65)***	1.21 (0.74-1.98)
Augmented oestrogen (group 2)	54 (2.1)	2481 (97.9)	0.45 (0.34-0.60)***	0.47 (0.34-0.63)***
**Age in years**				
Mean(SD)	73.2 (6.4)	60.7 (7.9)	1.19 (1.18-1.21)***	1.15 (1.14-1.17)***
**wCCI**				
Mean(SD)	3.8 (3.1)	1.4 (2.4)	1.27 (1.24-1.30)***	1.13 (1.10-1.16)***
**Income quintile, n(%)**				
Richest	47 (1.1)	4376 (98.9)	1(ref)	1(ref)
Rich	44 (1.3)	3346 (98.7)	1.22 (0.81-1.85)	1.14 (0.74-1.74)
Middle	75 (2.9)	2484 (97.1)	2.81 (1.95-4.06)***	1.64 (1.11-2.42)*
Poor	198 (9.5)	1886 (90.5)	9.77 (7.08-13.50)***	2.44 (1.71-3.47)***
Poorest	258 (11.8)	1937 (88.2)	12.40 (9.05-17.00)***	2.79 (1.96-3.98)***
**Education, n(%)**				
Tertiary	114 (2.1)	5228 (97.9)	1(ref)	1(ref)
Secondary	241 (3.5)	6615 (96.5)	1.67 (1.33-2.09)***	1.15 (0.90-1.47)
Primary	229 (10.3)	1988 (89.7)	5.28 (4.20-6.65)***	1.40 (1.07-1.81)*

Figure 2

393x152mm (600 x 600 DPI)

STROBE Statement—Checklist of items that should be included in reports of ***cohort studies***

	Item No	Recommendation
**Title and abstract**	1	(*a*) Indicate the study's design with a commonly used term in the title or the abstract. *"Association between pharmaceutical modulation of oestrogen in postmenopausal women in Sweden with death due to COVID-19 – a cohort study". Page 1, line 1-2*
		(*b*) Provide in the abstract an informative and balanced summary of what was done and what was found. *Page 3-4, line 28-62*
**Introduction**		
Background/rationale	2	Explain the scientific background and rationale for the investigation being reported. *Page 5, lines 75-90.*
Objectives	3	State specific objectives, including any prespecified hypotheses. *Page 5, lines 87-90*
**Methods**		
Study design	4	Present key elements of study design early in the paper. *Page 6-7, lines 91-134*
Setting	5	Describe the setting, locations, and relevant dates, including periods of recruitment, exposure, follow-up, and data collection. *Page 6, lines 96-114*
Participants	6	(*a*) Give the eligibility criteria, and the sources and methods of selection of participants. Describe methods of follow-up. *Page 6, lines 96-114*
		(*b*) For matched studies, give matching criteria and number of exposed and unexposed. *Not applicable.*
Variables	7	Clearly define all outcomes, exposures, predictors, potential confounders, and effect modifiers. Give diagnostic criteria, if applicable. *Page 7, lines 116-126*
Data sources/ measurement	8*	For each variable of interest, give sources of data and details of methods of assessment (measurement). Describe comparability of assessment methods if there is more than one group. *Page 6-7, lines 91-134*
Bias	9	Describe any efforts to address potential sources of bias. *Not applicable.*
Study size	10	Explain how the study size was arrived at. *Page 6-7, lines 91-134*
Quantitative variables	11	Explain how quantitative variables were handled in the analyses. If applicable, describe which groupings were chosen and why. *Page 6-7, lines 91-134*
Statistical methods	12	(*a*) Describe all statistical methods, including those used to control for confounding. *Page 7, lines 128-134*
		(*b*) Describe any methods used to examine subgroups and interactions. *Page 6-7, lines 91-134*
		(*c*) Explain how missing data were addressed. *Case only method.*
		(*d*) If applicable, explain how loss to follow-up was addressed. *Not applicable.*
		(*e*) Describe any sensitivity analyses. *Not applicable.*
**Results**		
Participants	13*	(a) Report numbers of individuals at each stage of study—eg numbers potentially eligible, examined for eligibility, confirmed eligible, included in the study, completing follow-up, and analysed. *Page 8, lines 135-142*
		(b) Give reasons for non-participation at each stage. *Not applicable.*
		(c) Consider use of a flow diagram. *Figure 1.*
Descriptive data	14*	(a) Give characteristics of study participants (eg demographic, clinical, social) and information on exposures and potential confounders. *Page 8, lines 135-142, Table 1*

For peer review only - http://bmjopen.bmj.com/site/about/guidelines.xhtml

		(b) Indicate number of participants with missing data for each variable of interest. *Table 1.*
		(c) Summarise follow-up time (eg, average and total amount). *Not applicable.*
Outcome data	15*	Report numbers of outcome events or summary measures over time. *Page 8, lines 144-158.*
Main results	16	(*a*) Give unadjusted estimates and, if applicable, confounder-adjusted estimates and their precision (eg, 95% confidence interval). Make clear which confounders were adjusted for and why they were included. *Page 8, lines 144-158. Figure 2.*
		(*b*) Report category boundaries when continuous variables were categorized. *Not applicable.*
		(*c*) If relevant, consider translating estimates of relative risk into absolute risk for a meaningful time period. *Page 8, lines 150-152.*
Other analyses	17	Report other analyses done—eg analyses of subgroups and interactions, and sensitivity analyses. *Page 8, lines 154-158.*
**Discussion**		
Key results	18	Summarise key results with reference to study objectives. *Page 9, lines 160-163.*
Limitations	19	Discuss limitations of the study, taking into account sources of potential bias or imprecision. Discuss both direction and magnitude of any potential bias. *Page 11, lines 209-217.*
Interpretation	20	Give a cautious overall interpretation of results considering objectives, limitations, multiplicity of analyses, results from similar studies, and other relevant evidence. *Page 11, lines 219-223.*
Generalisability	21	Discuss the generalisability (external validity) of the study results. *Page 9-10, lines 165-207*
**Other information**		
Funding	22	Give the source of funding and the role of the funders for the present study and, if applicable, for the original study on which the present article is based. *Page 12, lines 228-234.*

*Give information separately for exposed and unexposed groups.

Note: An Explanation and Elaboration article discusses each checklist item and gives methodological background and published examples of transparent reporting. The STROBE checklist is best used in conjunction with this article (freely available on the Web sites of PLoS Medicine at http://www.plosmedicine.org/, Annals of Internal Medicine at http://www.annals.org/, and Epidemiology at http://www.epidem.com/). Information on the STROBE Initiative is available at http://www.strobe-statement.org.

For peer review only
